# A patch of positively charged residues regulates the efficacy of clinical DR5 antibodies in solid tumors

**DOI:** 10.1016/j.celrep.2021.109953

**Published:** 2021-11-02

**Authors:** Gururaj Shivange, Tanmoy Mondal, Evan Lyerly, Sanchita Bhatnagar, Charles N. Landen, Shivani Reddy, Jonathan Kim, Britney Doan, Paula Riddle, Jogender Tushir-Singh

**Affiliations:** 1 Laboratory of Novel Biologics, Medical Microbiology and Immunology, University of California, Davis, Davis, CA 95616, USA; 2 Department of Medical Microbiology and Immunology, University of California School of Medicine, University of California, Davis, Davis, CA 95616, USA; 3 Department of Biochemistry and Molecular Genetics, University of Virginia School of Medicine, Charlottesville VA 22908, USA; 4 Undergraduate Research Program Volunteers, University of Virginia, Charlottesville VA; 5 Blavatnik Institute, Harvard Medical School, Boston MA; 6 Department of Gynecology and Oncology, University of Virginia; 7 University of Virginia Comprehensive Cancer Center, Charlottesville VA; 8 UC Davis Comprehensive Cancer Center, University of California School of Medicine, University of California, Davis, Davis, CA 95616, USA; 9 These authors contributed equally; 10 Lead contact

## Abstract

Receptor clustering is the first and critical step to activate apoptosis by death receptor-5 (DR5). The recent discovery of the autoinhibitory DR5 ectodomain has challenged the long-standing view of its mechanistic activation by the natural ligand Apo2L. Because the autoinhibitory residues have remained unknown, here we characterize a crucial patch of positively charged residues (PPCR) in the highly variable domain of DR5. The PPCR electrostatically separates DR5 receptors to autoinhibit their clustering in the absence of ligand and antibody binding. Mutational substitution and antibody-mediated PPCR interference resulted in increased apoptotic cytotoxic function. A dually specific antibody that enables sustained tampering with PPCR function exceptionally enhanced DR5 clustering and apoptotic activation and distinctively improved the survival of animals bearing aggressive metastatic and recurrent tumors, whereas clinically tested DR5 antibodies without PPCR blockade function were largely ineffective. Our study provides mechanistic insights into DR5 activation and a therapeutic analytical design for potential clinical success.

## In brief

Shivange et al. describe a negative regulatory epitope in the death receptor-5 ectodomain that limits apoptotic cytotoxicity. The negative regulatory domain co-targeting with dual-specificity antibody designs elicits sustained DR5 clustering and a superior anti-tumor response, explaining past clinical failures and revisiting future clinical strategies.

## Graphical Abstract

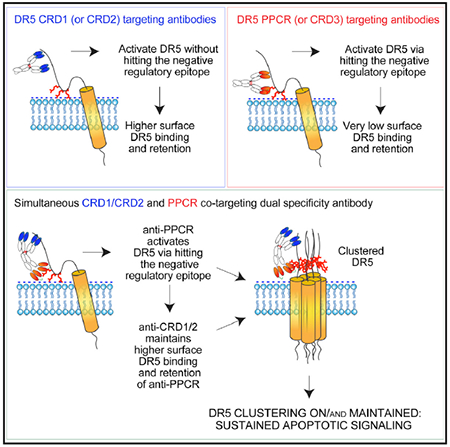

## INTRODUCTION

Death receptor-5 (DR5) belongs to the tumor necrosis factor alpha (TNF-α) superfamily, contains three external cysteine-rich domains (CRD1–CRD3), and activates apoptotic signaling outside of cells (Ashkenazi and Herbst, 2008). The DR5 ligand Apo2L and agonist strategies orchestrate apoptotic cytotoxicity by assembling an activated death-inducing signaling complex (DISC) under the lipid bilayer. This process requires higher-ordered clustering of the DR5 receptor (Hymowitz et al., 1999). However, the mechanism of external DR5 clustering initiation and maintenance has remained highly elusive. Multiple DR5 agonist antibodies, such as lexatumumab (humanized HGS-ETR2) from a Human Genome Sciences and Cambridge Antibody Technology collaboration (Johnson et al., 2003), AMG655 (conatumumab from Amgen; Kaplan-Lefko et al., 2010), KMTR2 from Kirin, Japan (Motoki et al., 2005), tigatuzumab (a humanized version of TRA-8 from Daiichi Sankyo; Forero-Torres et al., 2010), apomab from Genentech (Adams et al., 2008), etc., have been tested in the clinic for various solid cancer indications (Ashkenazi and Herbst, 2008; Shivange et al., 2018; Wajant, 2019). Unfortunately, all DR5 agonists failed in phase II clinical trials because of limited apoptotic cytotoxicity induction as a result of limited receptor clustering and maintenance (Ashkenazi, 2015; Forero-Torres et al., 2010).

By making use of Apo2L, a recent study has described the importance of the GXXXG motif in the transmembrane domain of DR5 and the autoinhibitory anti-clustering function of DR5 ectodomain (ECD) (Pan et al., 2019). These findings have challenged the long-standing view of DR5 activation. Because DR4 lacks the GXXXG motif, these results also point to subtle differences in the clustering mechanism of DR4 and DR5 by Ap02L (Pan et al., 2019), which maintains broad specificity against two receptors sharing 60% sequence similarity. Apo2L does so by binding DR4 and DR5 via combinations of multiple low-affinity interactions across various regions of the ECD, which collectively contribute to receptor clustering mechanisms (Hymowitz et al., 1999, 2000). Therefore, to identify DR5-selective negative regulatory and ECD auto-inhibitory residues, here we made use of high-affinity DR5-selective multiple clinical antibodies (alongside Apo2L) with the combination of functional receptor mutagenesis and *in vitro* clustering studies. We uncovered the negative regulatory function of a critical patch of positively charged residues (PPCR) in the highly variable CRD3 domain of DR5. Next, by analytically applying our mechanistic findings, we sought to investigate whether sustained antibody-mediated interference of negative regulatory PPCR residues would result in supremely effective DR5 clustering and apoptotic clustering to tumors.

## RESULTS

### Lexatumumab activated efficient cell death despite its unexpectedly low surface binding

**Figure 1.**
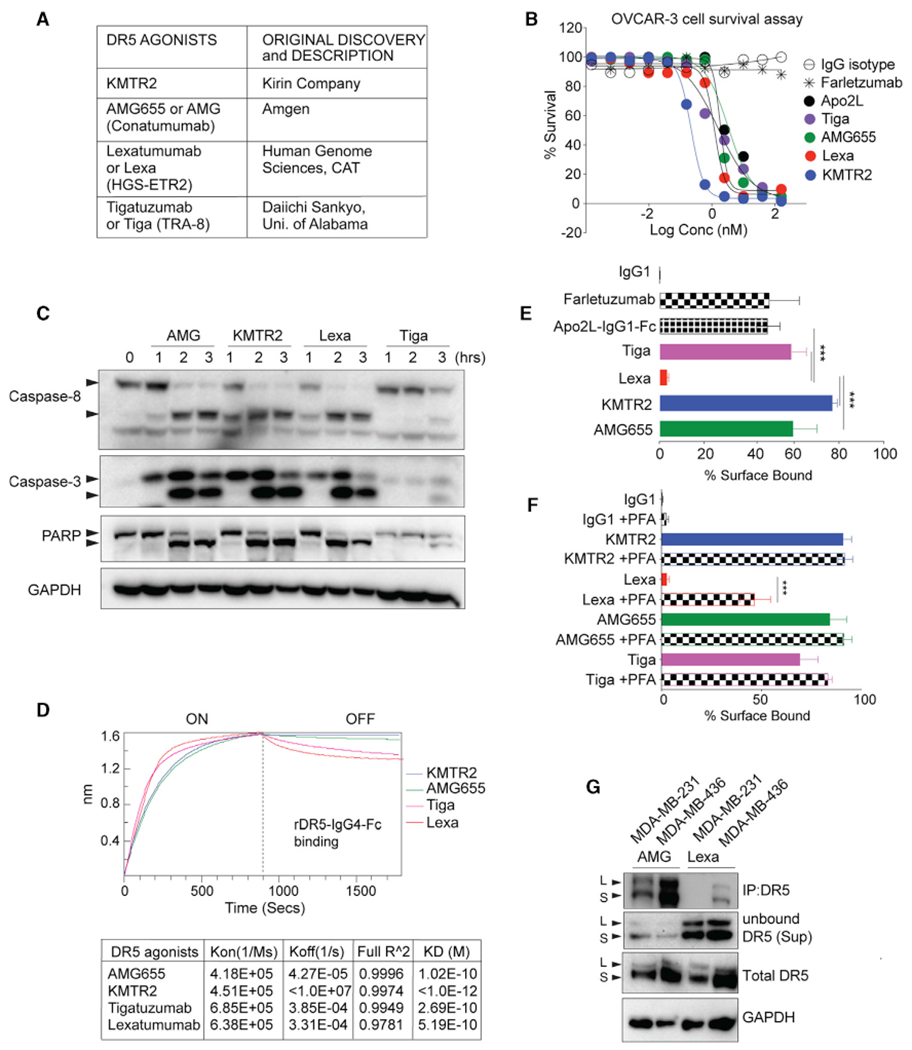
Lexa engages DR5 on the cell surface to a limited extent (A) List showing various DR5 antibodies used in the study. (B) Cell viability assays of the indicated DR5 agonists against OVCAR-3 cells (n = 3). (C) Tumor cells were treated with the indicated DR5 agonist antibodies for the indicated times. Lysates were analyzed for caspase-8, caspase-3, and PARP as apoptotic regulators using immunoblotting. (D) The binding kinetics of immobilized biotinylated recombinant DR5 (rDR5)-IgG4 against the indicated DR5 antibodies were measured using biolayer interferometry (BLI). (E) The percentage of indicated DR5 agonists bound on the tumor cell surface (n = 3). (F) Same as (E) except with or without crosslinking on ice with paraformaldehyde (PFA) (n = 3). (G) The indicated high (MDA-MB-436) and low(MDA-MB-231) DR5-expressing cells were treated with AMG655 or Lexa, followed by native immunoprecipitation using an anti-IgG1-Fc secondary antibody. Error bars in (E) and (F) represent SD (n = 3).

This study used various high-affinity pre-clinical and clinical DR5 agonist antibodies (Figure 1A) that activate caspase-8 and tumor cell death (Figures 1B and 1C). Surprisingly, despite high-affinity binding against recombinant DR5 *in vitro* (Figure 1D) and triggering cell death similar to other clinical DR5 agonist antibodies (Figures 1B and 1C), lexatumumab (Lexa) showed almost undetectable binding to surface DR5 (unless cross-linked) in cultured cells (Figures 1E, 1F, and S1A–S1D). Further, in native immunoprecipitation studies, compared with AMG655, significantly low DR5 was pulled down by Lexa (Figure 1G). To reconfirm limited Lexa binding to surface DR5 on tumor cells, we used a murine DR5-specific antibody, MD5-1. Unlike human DR5 agonist antibodies, MD5-1 activates tumor cell death of murine cells only when Fc crosslinked (Shivange et al., 2018; Figures S2A–S2C). Next we genetically engineered Lexa (and other DR5 antibodies, such AMG655 and KMTR2) with MD5-1 into dually specific antibodies as described earlier (Shivange et al., 2018; Figure S2D). We hypothesized that, because of its significantly lower surface DR5 binding on human tumor cells, unlike AMG655 and KMTR2, Lexa would not be an effective Fc-crosslinking partnerfor MD5-1 when murine and human tumor cells were co-cultured (Figure S2E). To test this, we treated human-murine tumor cell cocultures with MD5-1 containing dual-specificity antibodies (Figures S2D and S2E). MD5-1-AMG655 and MD5-1-KMTR2 completely eliminated human-murine tumor co-cultures, whereas MD5-1-Lexa was significantly less effective. (Figure S2F). These results physiologically reconfirmed the lower differential ability of Lexa (compared with other DR5 agonists) to engage DR5 on human tumor cells despite activating effective cell death (Figure 1B versus 1E).

### Lexa and apomab bind the highly variable CRD3 of DR5

**Figure 2.**
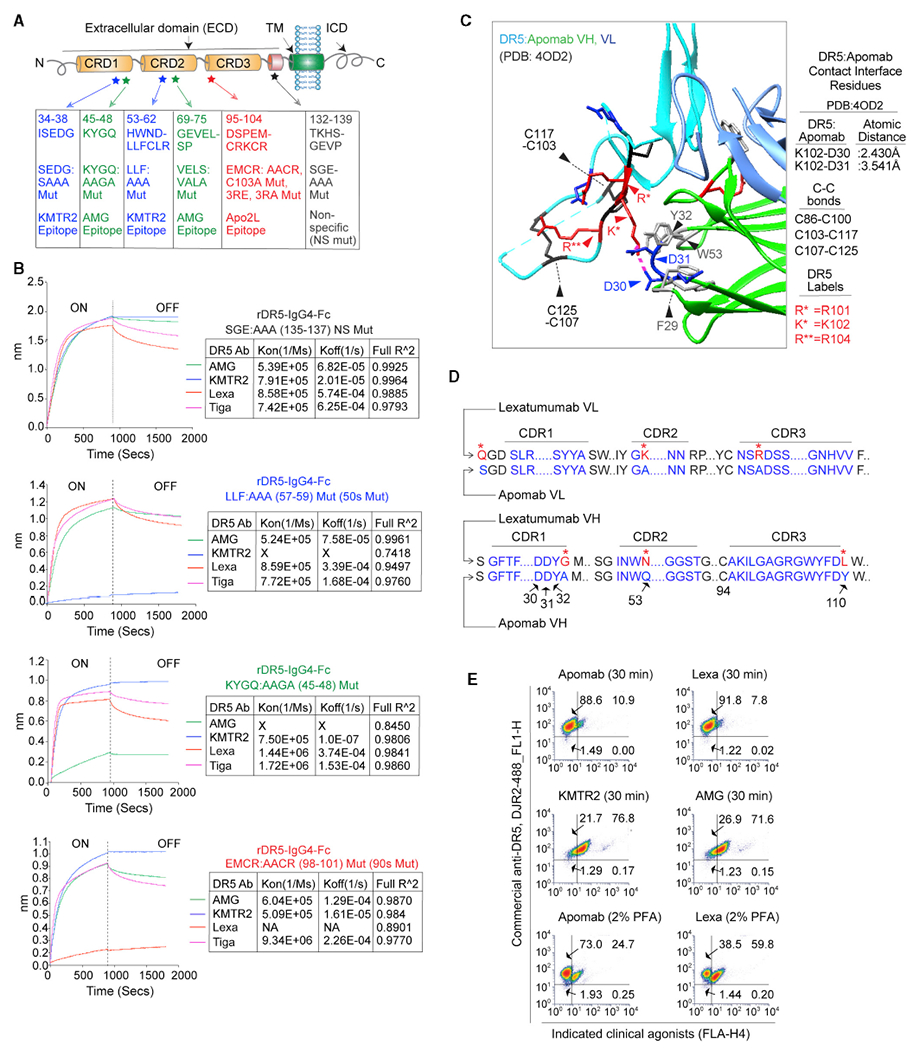
CRD3 targeting agonist antibodies engage surface DR5 to a limited extent (A) Schematic of DR5, showing binding epitopes of the indicated DR5 antibodies and Apo2L. CRD, cysteine-rich domain. (B) The binding kinetics of immobilized biotinylated rDR5 with the indicated mutations (top) against various DR5 agonist antibodies were measured using BLI. (C) Structural representation of the DR5-Apomap interface near the RKCR (101–104) DR5 residues (see also Figures 7B and 7C for Apo2L). Molecular interactions are shown with dotted pink lines. (DR5 backbone, cyan; apomab-VH/VL [variable heavy/variable light], green/sky blue). Red, blue, and gray highlight positively charged, negatively charged, and hydrophobic residues, respectively. The disulfide bonds (C86-C100, C103-C117, and C107-C125) are indicated as black sticks. Additional interface residue labels and details about atomic distances are shown outside of the interface box on the right. (D) Sequence of Lexa and apomab VH and VL CDRs. (E) The percentage of total surface DR5 was analyzed at the indicated times using a combination of commercial and indicated DR5 antibodies with or without crosslinking with PFA.

To further investigate the differentially low surface DR5 binding of Lexa, we analyzed the binding kinetics of various DR5 agonists using the ForteBio Octet system. Four different DR5 antibodies alongside Apo2L were tested against immunoglobulin G4 (IgG4) Fc wild-type DR5 and DR5 having mutations in the ECD. Key DR5 ECD mutation regions were chosen (Figure 2A) based on previously published and confirmed binding studies of KMTR2 (Tamada et al., 2015), AMG655 (Graves et al., 2014), and Apo2L (Hymowitz et al., 1999, 2000; Mongkolsapaya et al., 1999). An additional 132–139 region of DR5 ECD was used as a non-specific control (Figure 2A). When tested using biolayer interferometry (BLI), our binding results confirmed previously published studies (Figure 2B). We also confirmed these results with ELISA (data not shown). Interestingly, Lexa lost binding only against recombinant DR5 having mutations in the highly variable CRD3 (residues 98–101, EMCR-AACR substitution) domain (Figure 2B). Notably, despite numerous CRD1- and CRD2-tar-geting DR5 agonist antibodies, apomab is the only known (or published) CRD3 targeting DR5 agonist antibody (Adams et al., 2008). Similar to Lexa, apomab is an effective apoptosis activator (Adams et al., 2008). The DR5 binding interface of apomab VH (variable domain of heavy chain) contains a loop of two critical negatively charged aspartate D30 and D31 residues (structurally stabilized by a hydrophobic residue core of F29, Y32, and W53), which also form a salt bridge with the DR5 PPCR, specifically with the K102 (D30–K102, 2.430 Å; PDB: 4OD2) of the RKCR residues (Figure 2C). Interestingly, Apomab is ~95% similar to Lexa, with six key substitutions in complementarity-determining regions (CDRs) (Figure 2D; sequence source, PDB: 4OD2). Strikingly, two critical residue differences in Lexa VH CDRs are right next to structure-stabilizing Y32 (A33G) and W53 (Q54N) residues. Further, the RKCR residues are next to the described E99 and M101 residues of 90’s loop region of DR5 (Hymowitz et al., 1999), which are critical for interactions with the Y216 and Q205 residues of Apo2L (Hymowitz et al., 1999, 2000; Mongkolsapaya et al., 1999) during receptor activation and apoptotic signaling. Furthermore, Apo2L also cooperatively enhances AMG655-mediated DR5 activation (Graves et al., 2014) but interferes with Lexa-mediated DR5 apoptotic signaling (Shivange et al., 2018). These results indicate a shared DR5 activation epitope by Lexa, apomab, and Apo2L. When tested using flow cytometry studies, apomab’s tumor cell surface DR5 binding profile matched very closely to Lexa rather than KMTR2 or AMG655 (Figure 2E). Thus, we used Lexa as a CRD3 engaging antibody next to CRD1/2 binding antibodies for follow-up investigations. In line with studies published previously (Hymowitz et al., 1999, 2000), Apo2L lost some degree of binding to all generated DR5 ECD mutants, confirming the importance of its multiple low-affinity interactions throughout the DR5 ECD for DR5 clustering (Figure S3A).

### Differential receptor clustering profile of DR5 agonists

**Figure 3.**
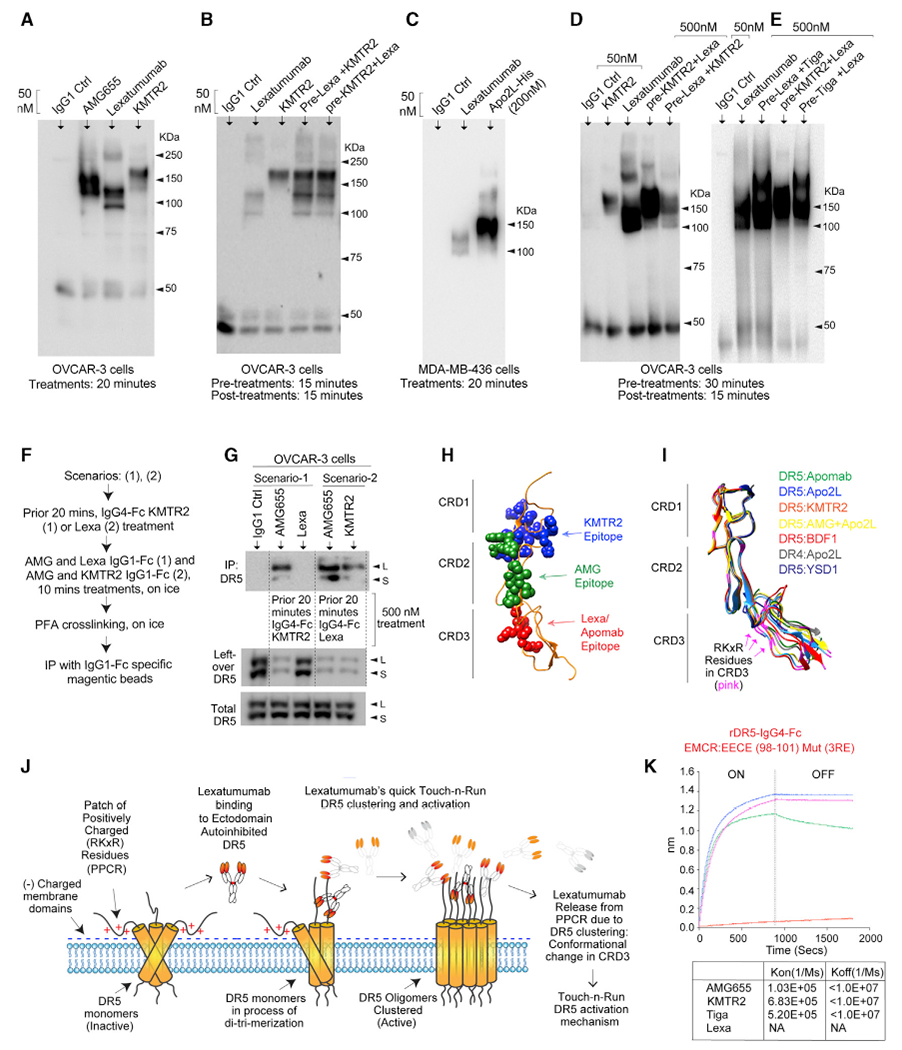
Lexa binds PPCR in the highly variable CRD3 domain of DR5 (A–E) Tumor cell lysates after the indicated DR5 agonists treatments (top), concentration (top), and time (bottom) were analyzed with non-reducing denaturing clustering assays using DR5 immunoblotting. (F) Schematic of the experiment described in (G). (G) Upon the indicated treatment, OVCAR-3 cells lysates were immunoprecipitated as shown. Unbound and total lysates from the latter were also analyzed using DR5 immunoblotting. (H) Colored spheres indicate distinct epitopes of the indicated DR5 agonists in 3 different CRDs. (I) A ribbon trace backbone of DR5 bound to the indicated DR5 agonist antibodies or natural ligands. See text for various PDB IDs. RKxR harboring CRD3 shows the highest variability. (J) Cartoon of the native autoinhibited DR5 ECD, showing electrostatic interaction of the PPCR motif (in CRD3) with negatively charged membrane domains. Lexa binding to the PPCR results in conformational change and release. (K) The binding kinetics of immobilized biotinylated 3RE mutant rDR5 against the indicated antibodies were measured using BLI.

Unlike trimeric Apo2L binding to trimeric DR5, agonist antibodies are bivalent. Hence, bivalent DR5 antibody-mediated clustering and mechanisms have remained untested. To investigate why selective binding of Lexa interferes with its interactions with cell surface DR5, we performed non-reduced (without β-mercaptoethanol [BME] in lysates) denaturing clustering assays (Valley et al., 2012). Various DR5 antibodies showed distinct clustering profiles (from each other) when treated side by side, supporting their potentially different mechanism of DR5 activation. In particular, only Lexa-treated lysates showed a lower clustering profile with a distinct band of ~100 kDa (Figures 3A–3D and S3B). Neither AMG655 nor KMTR2 generated this particular lower clustering band (Figures 3A–3D). Next we analyzed DR5 clustering after pre-KMTR2 (or pre-Lexa) treatment. The mixed DR5 clustering profile of both antibodies was evident on immunoblotsat 50 nM non-DR5-saturating concentrations, regardless of the order in which two DR5 antibodies were added to cells (Figures 3B and S3B). At 500 nM DR5 saturating concentration, 30-min pre-treatment with KMTR2 (or tigatuzumab [Tiga]) inhibited generation of a Lexa-driven DR5 clustering profile; particularly, the distinct band of ~100 kDa was not evident (Figures 3D and 3E). On the other hand, Lexa pre-treatment did not affect the Tiga clustering profile (Figures 3D and 3E, third and fourth lanes). Because the 500-nM KMTR2 preincubation step represents saturated DR5 clustering, these results indicate Lexa’s inability to engage clustered and activated DR5. In support of this, KMTR2 pre-treatment blocked DR5 pull-down by Lexa, whereas KMTR2 and AMG655 maintained binding to native and activated DR5 (Figures 3F, 3G, S3C, and S3D). Thus, Lexa preferentially engages ECD-autoinhibited (a native form of receptor lacking straight activated columns) as described (Pan et al., 2019), but not the activated clustered DR5. These results also support the rapid release kinetics mechanism (Touch-n-Run) of Lexa following DR5 activation (Figure 1).

To further explore Lexa’s inability to engage activated DR5, we generated a ribbon trace backbone of DR5 (and DR4) bound to the ligand Apo2L (PDB: 1D0G); to highly agonistic AMG655 (PDB: 4N90), KMTR2 (PDB: 3X3F), and apomab (PDB: 4OD2) antibodies; or to limited agonistic BDF1 (PDB: 2H9G) and YSD1 (PDB: 1ZA3) antibodies (Figures 3H and 3I). Structurally, CRD1 and CRD2 maintained a similar conformation regardless of being in interface with agonist or non-agonist antibodies (Figure 3I). On the other hand, DR5 exhibits significant variations in CRD3 (Figure 3I), which may adopt an ensemble of conformations. One such confirmation could be the ECD autoinhibited form of DR5 or one where ECD access in the absence of ligand or agonist antibody binding is incompatible with formation of the multimeric complex required for signaling (Pan et al., 2019). Because Lexa selectively binds to the highly variable DR5 ECD in the CRD3 region (Figures 2B, 3H, and 3I), one variable ensemble of DR5 conformation potentially represents the ECD-autoinhibited form of DR5, as described previously using nuclear magnetic resonance structure studies (Pan et al., 2019). Hypothetically, upon DR5 activation, the Lexa epitope could potentially change structurally (epitope burial in the complex) with the higher-order clustered DR5 column assembly, resulting in its rapid release (Figure 3J).

### Charge substitution of the PPCR increases extrinsic apoptotic signaling

**Figure 4.**
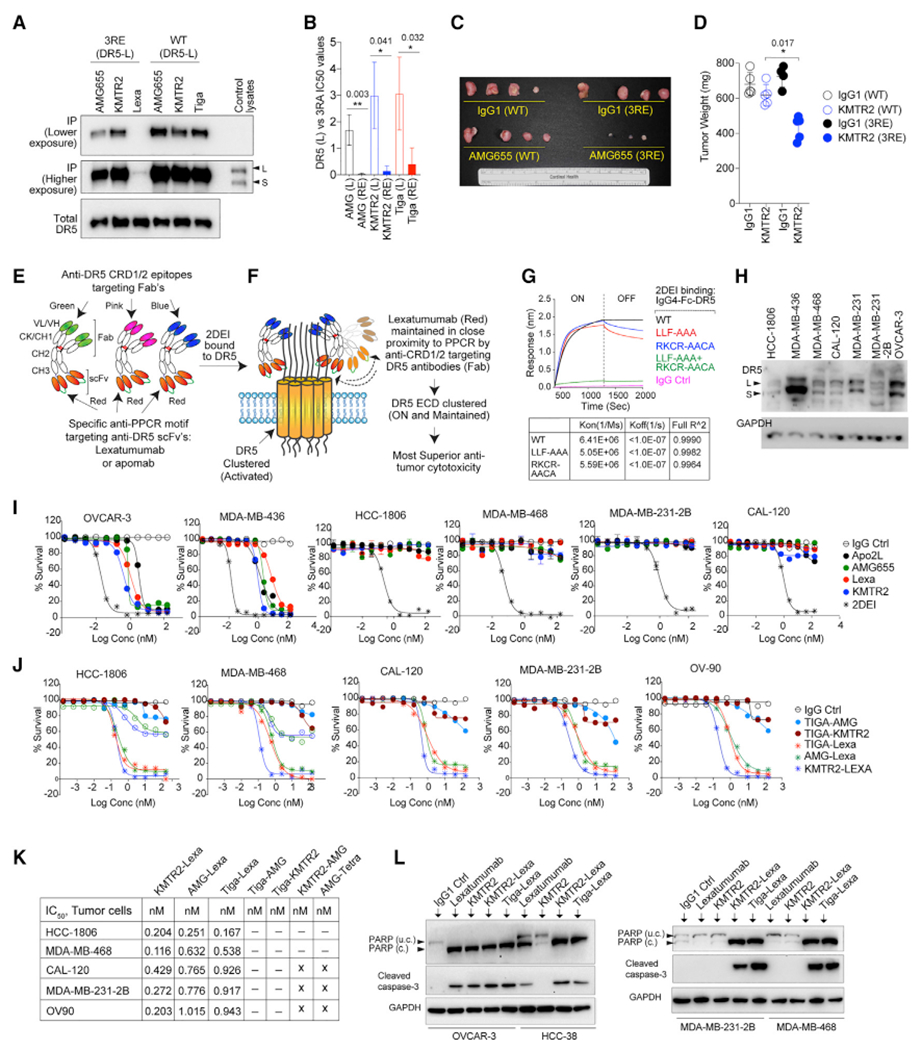
Genetic construction and working mechanism of the 2DEI antibody (A) WT (L) and 3RE-Mut (L) DR5 stable cells were subjected to immunoprecipitation as indicated. (B) Fold change in IC50 values in 3RE-Mut (L) DR5 stable cells versus WT cells after the indicated antibodies (n = 3). (C and D) DR5 knockout MDA-MB-231 (C) and HCC-1806 (D) cells stably expressing WT-DR5(L) and DR5-3RE were grafted with orthotopic tumors in the mammary fat pad. Randomly selected tumor-bearing animals were injected intraperitoneally (i.p.) with IgG1 and KMTR2 (100 μg) every third day. Harvested tumors were quantified. (E and F) Genetic construction schematic of the dual-specificity 2DEI antibody, where a CRD1- or CRD2-targeting bispecific partner was engineered to enhance Lexa (or apomab) engagement against the PPCR motif before and after higher-order DR5 clustering. (G) The binding kinetics of immobilized biotinylated rDR5 (indicated single and double mutations; Figure 2A) against 2DEI were measured using BLI. (H) DR5 (S) and DR5 (L) expression in various human TNBC cell lines. (I) The indicated ovarian and TNBC cell lines were analyzed in cell viability assays with the indicated clinical DR5 agonists and 2DEI antibody (KMTR2-Lexa). (J) Same as (E), except additional 2DEI antibodies (generated with AMG655 [AMG]-Lexa and Tiga-Lexa), and random bispecific antibodies were used against highly resistant TNBC cell lines. (K) IC_50_ values from (F). (L) Immunoblotting of PARP and caspase-3 from the indicated cell lysates after the indicated treatments. Error bars in (B) and (D) represent SD and SEM, respectively (n = 3).

The DR5 residues in the highly variable DR5 CRD3, next to Apo2L’s Y216 and Q205 binding region (Hymowitz et al., 1999), contains an externally exposed PPCR (RKCR: 101–104; Figures 3I and 3J). C103 in RKCR is the third cysteine that forms a disulfide bond with the fifth cysteine (C117) of CRD3 to generate rigid body looping (Hymowitz et al., 1999, 2000; Mongkolsapaya et al., 1999). The DR5 ECD is autoinhibitory by a mechanism that potentially involves steric hindrance (Endres et al., 2013) or charge-based receptor looping away from other DR5 molecules (Endres et al., 2013; Pan et al., 2019). Thus, we next wanted to determine whether the PPCR is critical for Lexa binding and works similar to the previously described kinase domain harboring KKIK residues of epidermal growth factor receptor (EGFR) for receptor activation (Figures 2I and 2J; Endres et al., 2013). We hypothesized that stabilized electrostatic interactions of the PPCR with negatively charged membrane domains (Lin et al., 2017) or negatively charged membrane receptors potentially generate and contribute to pre-ligand autoinhibitory ECD loop formation. The latter constrains neighboring DR5 receptors to initiate oligomerization and higher-order clusters (Pan et al., 2019; Figure 3J). To test this hypothesis, we generated charge and disulfide bond substituted recombinant DR5 harboring 3RE (RKCR-EECE), 3RA (RKCR-AACA), and C103A (RKCR-RKAR) mutations. The binding studies confirmed significant and complete loss of only Lexa and apomab against 3RE and C103 mutants, respectively (Figures 3K and S3E–S3G). To test this *in vivo*, we generated endogenous DR5 knockout MDA-MB-231 triple-negative breast cancer (TNBC) cells and stably infected them with 3RE and 3RA DR5(L) mutant lentiviral vectors (Figures 4A and S4A–S4C). Strikingly, all tested DR5 antibodies (except Lexa) showed significantly higher caspase-8 and cell-killing activity against 3RE and 3RA MDA-MB-231 cells and tumors expressing similar levels of DR5(L) (Figures 4B–4D and S4D–S4G). When tested, DR5 clustering generated by AMG655 and KMTR2 was higher against 3RE and 3RA stable cells than wild-type (WT) DR5(L) cells (Figures S5A–S5F). Thus, PPCR charge substitution (3RA and 3RE mutants) and charge neutralization (by Lexa) serve to enhance DR5 clustering and activation. To determine whether potential electrostatic looping of the PPCR away from the receptor serves the negative regulatory role of clustering autoinhibition in native DR5, additional structural studies are needed. Nonetheless, these results indicate the negative apoptotic regulatory function of PPCR.

### Generation and activity of the 2DEI antibody

Because the PPCR engaging Lexa incorporates “touch and run” DR5 binding and activation kinetics (Figures 1 and 3J), next we investigated the possibility of increasing sustained interference against PPCR autoinhibition function. To this end, we genetically linked Lexa with CRD1- and CRD2-engaging DR5 antibodies to maintain Lexa’s sustained interference with the PPCR. We named this the dual DR5 ECD inhibition (2DEI) targeting antibody strategy (Figures 4E and 4F). The binding data confirm the 2DEI (KMTR2-Lexa) ability to simultaneously engage two different DR5 epitopes (Figure 4G). A loss of binding function mutation of the KMTR2 epitope (LLA-AAA) still maintained 2DEI-DR5 interactions via Lexa and vice versa (Figure 4G). 2DEI lost binding only when both epitopes were mutated (Figure 4G, green line). When tested against multiple TNBC cell lines (having variable DR5 expression), the 2DEI antibody had the highest cytotoxicity, whereas clinical antibodies were ineffective against multiple TNBC cell lines (Figures 4H and 4I). Strikingly in all cases, only the PPCR targeting Lexa containing bispecific combinations (KMTR2-Lexa, AMG655-Lexa, and Tiga-Lexa) showed the highest cytotoxic activity, caspase-3 and poly(ADP-ribose) polymerase (PARP) cleavage (Figures 4J–4L, S5G, and S5H). Similar results were seen when Lexa scFv (single-chain variable fragment) was replaced with apomab scFv (Figures S6A and S6B). Random bispecific antibody combinations without the capability to co-engage the PPCR epitope were only somewhat effective against highly resistant TNBC cells (Figures 4J–4L, S5G, S5H, and S6B).

**Figure 5.**
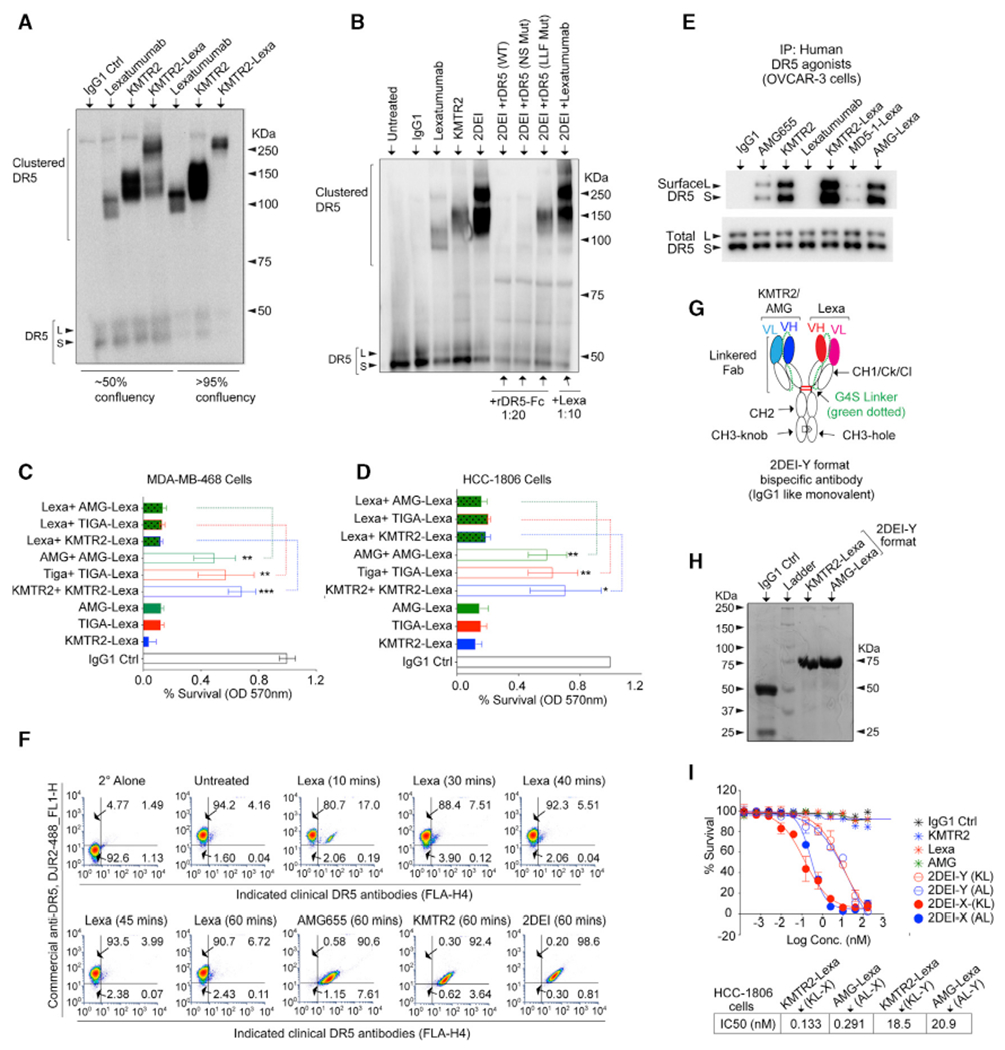
Enhanced DR5 clustering and cytotoxicity by 2DEI is independent of the bispecific antibody format (A) The *in vitro* DR5 clustering profile after the indicated antibody treatments with the indicated cell confluences of OVCAR-3 cells in non-reducing denaturing gels. (B) OVCAR-3 tumor cells were treated with the indicated antibodies for 20 min. In additional sets, tumor cells were pre-incubated (10 min) with 20-fold recombinant DR5 proteins (WT DR5, NS [non-specific] mutant DR5, and LLF mutant DR5; Figure 2A) or 10-fold Lexa IgG1 before 2DEI antibody treatment. Cellular lysates were analyzed in non-reducing denaturing gels and immunoblotted for DR5. (C and D) Percent survival analysis of MDA-MB-468 and HCC-1806 cells treated with three different 2DEI antibodies. In parallel experiments before treatment with three different 2DEI antibodies, corresponding monoclonal IgG1s (such as KMTR2, AMG655, and Tiga) were added in one experimental set, or Lexa was added for the second experimental set (n = 3). (E) Native immunoprecipitation of DR5 with the indicated DR5 agonists, 2DEI, and random bispecific antibodies using OVCAR-3 cells. (F) The percentage of total surface DR5 was analyzed using flow cytometry at the indicated times using a combination of commercial and indicated DR5 agonist antibodies (n = 3). (G) Genetic construction schematic of the VL-VH linked Y-format IgG1-like, monovalent, dual-specificity 2DEI antibody (2DEI-Y). (H) A reducing gel of Y-format 2DEI antibodies. Only the IgG1 control contains a VL chain on the gel. (I) Cell killing of HCC1806 cells in the presence of bivalent bispecifics (2DEI-X) or monovalent bispecifics (2DEI-Y) and the indicated regular bivalent monospecific antibodies. Error bars in (C), (D), and (I) represent SD (n = 3).

When tested using clustering assays, the 2DEI antibody completely reshuffled the Lexa clustering pattern to higher molecular weights regardless of tumor cellular confluency (Figures 5A and 5B). Unlike preincubation of recombinant DR5 proteins, 10-fold Lexa preincubation did not change the 2DEI-mediated clustering profile (Figures 5B) or cell death (Figures 5C and 5D). On the other hand, 10-fold preincubation of KMTR2 (or AMG655) significantly interfered with 2DEI-mediated gain of apoptotic function (Figures 5C and 5D). As expected, 2DEI antibodies pull down the highest surface DR5 in native immunoprecipitation (Figures 5E and S6C), confirming their ability to maintain Lexa close to the PPCR epitope for the longest period of time in higher-order complexes. Using combinatorial commercial and clinical DR5 antibodies, we also tested differential DR5 surface presence when treated with the 2DEI antibody using a flow cytometry early time course experiment. We did not observe significant overall DR5 internalization by Lexa compared with other clinical DR5 agonists or the 2DEI antibody (Figure 5F). Thus, enhanced apoptosis by the 2DEI antibody is due to mechanistic targeting of the negative regulatory PPCR domain. These results further support Lexa-IgG1’s inability to remain bound to DR5 when activated.

Next, we wanted to determine whether enhanced death agonism by the 2DEI antibody depended on a particular bispecific antibody format. To this end, we generated dual-specificity 2DEI antibodies by genetically linking the 3′ end of the VL chain with the 5′ end of VH chains using flexible G4S linkers (Figure 5G), as described earlier by our group (Shivange et al., 2018). We named this new bispecific antibody Y-2DEI because it resembles a Y-shaped IgG1 (Figure 5G) and is similar to the knob-hole-based CrossMab antibodies of Genentech (Ridgway et al., 1996; Schaefer et al., 2011). Because light and heavy chains are linked genetically in a Y-2DEI antibody, no VL was evident under reducing conditions (Figure 5H). Strikingly, monovalent bispecific Y-2DEI antibodies were also more effective at killing tumor cells than bivalent monospecific clinical antibodies (Figure 5I).

### Anti-tumor efficacy of 2DEI in solid tumors

**Figure 6.**
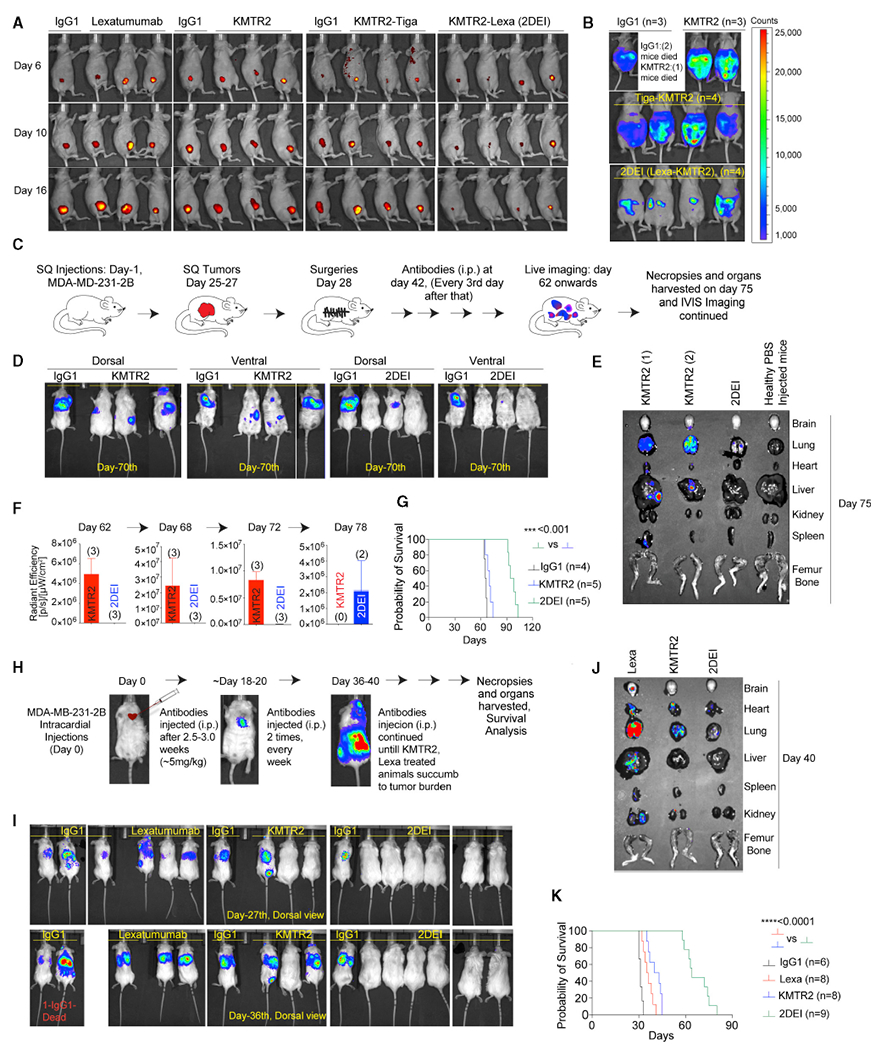
Anti-tumor efficacy of the 2DEI antibody (A) Mammary fat pad HCC-1806 tumor-bearing NSG mice were injected i.p. with the indicated antibodies for the indicated days. Animals were live imaged. (B) Intraperitoneal OV90 ovarian tumor-bearing animals were injected i.p. with 2DEI and random bispecific combinations along with KMTR2. All animals were live imaged when 2 of 3 IgG1-treated animals became moribund. (C and D) NSG animals were grafted with luciferase (Luc+) MDA-MB-231 cells to generate primary subcutaneous tumors. When tumors reached ~700 mm^3^ (~28 days), surgery was performed. After a 2-week recovery after surgery, animals were injected i.p. with IgG1, KMTR2, and 2DEI (100 μg) every third day, and tumor-bearing animals were live imaged on the indicated day (see also Figure S6D). (E) Same as (D), except animal necropsies were recovered (on day 75) from representative animals and analyzed by fluorescence imaging for detailed organ-specific tumor load (see also Figure S6E). (F) The indicated days on the top of graph represent the assay time of accumulated luciferase signal (radiant efficiency) from animals after KMTR2 and 2DEI antibody injections. (G) Kaplan-Meier plot depicting survival from (C) (n = 6–9 mice). (H) Highly metastatic MDA-MB-231-2B TNBC cells were injected intracardially to generate highly aggressive metastatic tumors that spread in the lungs and peritoneal cavity over 4–5 weeks. (I) Randomly selected metastatic tumor-bearing animals from (H) were treated (100 μg, 2 times a week) with the indicated antibodies after 2 weeks of intracardiac injection of tumor cells. Animals were live imaged on the indicated day (see also Figure S7A). (J) Same as (I), except animal necropsies were performed (on day 40) on representative animals and analyzed by fluorescence imaging for detailed organ-specific tumor load (see also Figure S7B). (K) Kaplan-Meier plot depicting survival from (I) (n = 6–12). Error bars in (F) represent SD (n = 2–3).

To confirm the apoptotic transition threshold’s lowering because of the loss of PPCR function *in vivo*, we used low-DR5-expressing, red fluorescent protein (RFP)-stable HCC-1806 (Figure 4H) breast fat pad tumor xenografts. Lexa, KMTR2, and Tiga-KMTR2 were completely ineffective, whereas 2DEI (KMTR2-Lexa) effectively eliminated these tumors (Figure 6A). Similarly, superior efficacy results were seen in intraperitoneal OV90 cells (ovarian) tumors (Figure 6B) and MDA-MB-468 tumors (Figure S6D). Because individuals with primary TNBC respond effectively to surgery and chemotherapy, we generated recurrent tumors in female non-obese diabetic (NOD) severe combined immunodeficiency (SCID) gamma (NSG) mice after surgically removing subcutaneous tumors (Figure 6C) for testing in more clinically relevant settings. After surgery, spontaneous secondary TNBC tumor-bearing animals treated with the 2DEI antibody had a significantly reduced tumor burden and survived significantly longer (Figures 6D–6G, S6E, and S6F).

Next we tested an aggressive experimental metastatic tumor model (EMTM) originating from the TNBC brain metastatic derivative of human mammary cells called 231-2B cells (Jenkins et al., 2005; Tominaga et al., 2015). These cells form highly aggressive tumors and have shown metastases comparable with human TNBC when injected into female NSG mice via the intracardiac route (Figure 6H; Przanowski et al., 2020). Randomly selected EMTM animals treated with clinical Lexa and KMTR2 antibodies had tumor burdens similar to IgG1-treated animals and died before day 45 (Figures 6I–6K). 2DEI-injected mice had reduced tumor loads, as seen by necropsies (Figures 6I, 6J, S7A, and S7B), and survived an average of 65 days (Figures 6K) with insignificant changes in weight (Figures S7C). Similar higher 2DEI efficacy results were seen in spheroid cultures derived from individuals with TNBC and tumor breast fat pad xenografts derived from affected individuals (Figures S7C–S7E).

## DISCUSSION

By making use of multiple clinical DR5 antibodies and Apo2L, here we discovered the negative apoptotic regulatory role of the PPCR in the highly variable CRD3. Mutational substitution of the PPCR significantly increased tumor cell death, implying a potential role of the PPCR in relaxing the autoinhibitory ECDs of DR5 receptors. Because variable protein domains (such as CRD3) are known to undergo the most considerable conformational changes upon assembly into a complex, our findings of Lexa’s potential “touch and run” working mechanism agree with the literature (Boehr et al., 2009). Upon Lexa binding, the surface-exposed PPCR with the autoinhibited ECD is potentially buried in the clustered activated columns of the oligomerized receptor because of conformational clustering (Pan et al., 2019). A similar conformational looping mechanism exists for EGFR activation (Endres et al., 2013). The PPCR is identical to the described positively charged patch of KKIK residues in the kinase domain of EGFR, approximately 30 amino acids (aa) away from the juxtamembrane segment. It is involved in looping to interact with the negatively charged plasma membrane at the cytosolic side of cells (Endres et al., 2013). The binding of epidermal growth factor (EGF) to EGFR brings a conformational change in the receptor, reliving electrostatic interactions of KKIK residues with the membrane to allow kinase domain dimerization and activation (Endres et al., 2013). Cancer cells are indeed known to lose polarity because of differential and increased distribution of negatively charged phosphatidylserine, phospholipids, and phosphoproteins on the extracellular side of the membrane (Bernardes and Fialho, 2018). Nearly all highly metabolically active cancer cells generate a significant amount of lactate as mobile anions to orchestrate a net negative cell surface charge (Chen et al., 2016). These findings point toward potential stabilized electrostatic interactions of the PPCR with the negatively charged membrane domains (Lin et al., 2017). Alternatively, negatively charged membrane receptors or additional steric hindrance mechanisms could be involved in orchestrating the ECD autoinhibitory function to constrain neighboring DR5 receptors to initiate oligomerization, as described earlier (Pan et al., 2019).

**Figure 7.**
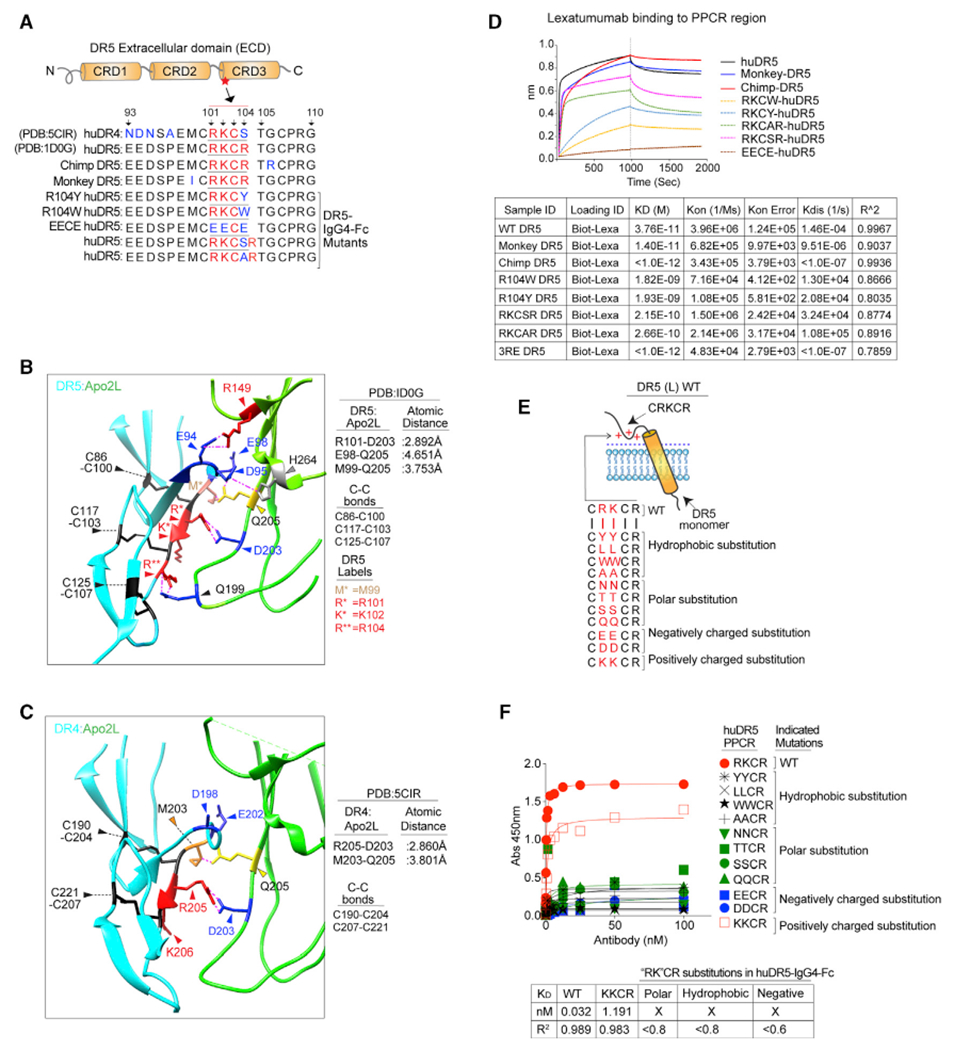
Apo2L also engages humans and non-human primate conserved RKcR residues (A) Detailed amino acid sequence around DR5 PPCR (red) in humans and non-human primates. (B and C) Cartoon representation of the DR5-Apo2L and DR4-Apo2L interface near PPCR motif. Molecular interactions are shown with dotted pink lines. DR5 backbone, cyan; Apo2L, green; Apomab-VH/VL, green/sky blue. Red, blue, and black highlight positively, negatively, and cysteine residues, respectively. Additional interface residues are shown with other colors. Details about disulfide bonds and atomic distances are shown outside of the interface boxes on the right. (D) Various generated recombinant IgG4-Fc human DR5 with mutations/insertions (in PPCR residues from A) were analyzed for binding kinetics of Lexa using BLI. (E) Cartoon schematic showing the potential ECD autoinhibitory PPCR (RKCR) of DR5. Various recombinant IgG4-Fc human DR5-harboring double mutations were generated by replacing positively charged “RK” residues as indicated. (F) Binding affinity of Lexa-IgG1 against various “RK” mutant DR5 molecules (from E) was determined by ELISA.

Lexa and apomab, engaging the PPCR and neighboring cysteine residues in DR4 and DR5, remain broadly preserved in humans and non-human primates (Figures 7A; Hymowitz et al., 2000; Ramamurthy et al., 2015). Consistent with previous crystal structure studies, among the various low-affinity interactions distributed evenly from the upper to the lower tip of the DR5 trimer (Cha et al., 2000; Hymowitz et al., 1999; Mongkolsapaya et al., 1999), Apo2L also makes critical contacts near the PPCR region of DR5 and DR4 (Figures 7B and 7C). Thus, with regard to the negative regulatory role of the PPCR, our findings explain why nature has selected low-affinity Apo2L as a common DR4/DR5 ligand. Specifically, just above the PPCR, Q205 of Apo2L interacts hydrophobically with E98 and M99 of DR5 and within the PPCR, D203 of Apo2L forms ionic salt bridge interactions with R101 (Figure 7B; Hymowitz et al., 1999, 2000). The ionic interacting distances between Apo2L D203 against R101 (DR5) and R205 (DR4) are only 2.892 Å and 2.860 Å respectively. In support of this, previous studies have described apoptotic competition between the PPCR targeting Lexa with Apo2L but not with the non-PPCR targeting AMG655 (Graves et al., 2014; Shivange et al., 2018).

KMTR2, AMG655, YSD1, and BDF-1 are known to target CRD1 and CRD2 of DR5, whereas apomab is a known CRD3-targeting molecule (Adams et al., 2008). Instead of R101, apomab forms salt bridge interactions with the K102 residue of the PPCR via the negatively charged D30 and D31 residues (Figure 2C). Despite the significant high similarity with Lexa, apomab (PDB: 4OD2) contains substituted different residues in the CDRs of VL and VH chains (Figure 2D). It remains to be established whether these substitutions (in particular Q/N) have differential propensities to alter specific side-chain bonding (Vasudev et al., 2012) of nearby hydrophobic residues to influence the D30 and D31 residue interactions with K102 (of RKCR). Additional DR5 structural studies are needed with Lexa. Unlike KMTR2, similar to Lexa, apomab showed significantly reduced binding to surface DR5 on cells and 3RE mutant recombinant DR5 *in vitro* (Figure 2E). Furthermore, saturation mutagenesis of RKCR with hydrophobic, polar, and negatively charged amino acids eliminated Lexa binding to DR5 (Figures 7D–7F), and all non-PPCR-targeting antibodies gained cytotoxic function after positive charge substitutions (Figures 4A–4D). These results strongly argue that the proposed positive charge-based anti-clustering ECD autoinhibitory mechanism is broadly applicable to improve DR5 clustering and activation by all DR5 agonists. Similar to Lexa, apomab has also failed to move beyond phase II trials (Ashkenazi, 2015). Thus, co-targeting anti-PPCR antibodies with CRD1- or CRD2-engaging antibodies via dual specificity could potentially move death receptor agonism beyond phase II trials.

How do 2DEI antibodies achieve complete cell killing despite significantly lower DR5 expression in CAL-120, HCC1806, and MDA-MB-468 cells? Sustained surface DR5 signaling has been shown to restore sensitivity to DR5 therapy in resistant cells (Jin et al., 2004). Therefore, the 2DEI antibody’s potential to remain bound to higher-order DR5 complexes and to increase the surface lifetime of Lexa close to the PPCR epitope is well suited to maintain persistent DR5 activation, despite its lower expression. A significant proportion of individuals with TNBC and colon cancer express elevated DR5 levels (Camidge et al., 2007; Forero-Torres et al., 2010). Despite the latter, phase II trials of Tiga and apomab as a single agent or a combination of nab-paclitaxel have proven disappointing against TNBC and advanced colon cancer (Camidge et al., 2007; Forero-Torres et al., 2015). If 2DEI can improve survival of individuals with TNBC and colon cancer, then it needs to be used in clinical trials. We provide mechanistic insights into limited tumor cytotoxicity by clinical DR5 agonists. Along with factors regulating the varying expression of DR5 (Ashkenazi, 2015), apoptotic regulators (Spencer et al., 2009), or negatively charged sialylated O-linked glycans within DR5 (Wagner et al., 2007), heterogenous tumor cells may also exploit the described mechanism of differential negative charge distribution across the selective membrane domains to interfere with receptor clustering, the apoptotic threshold, and clinical resistance.

### Limitations of study

Our *in vitro* results supported with *in vivo* animal studies suggest that dual engagement of DR5 via an anchor domain in the combination of PPCR domain could be involved in effectively overcoming ECD autoinhibition, as described previously (Pan et al., 2019). However, if PPCR-based mechanism alone is responsible, similarly to the previously described EGFR electrostatic looping-based ensemble of conformation (Endres et al., 2013) to constrain DR5 clustering, is still unknown. Future hydrogen-deuterium exchange (HDX) mass spectrometry and cryoelectron microscopy (cryo-EM) studies with 3RA (or 3RE) mutant DR5 are required to reveal the molecular mechanisms of PPCR-mediated ECD looping and its underlying effects on receptor clustering and activation. Furthermore, if additional unidentified binding partners (independent of membrane charge) contribute to steric mechanisms to autoinhibit DR5 ECD, then additional genome-wide CRISPR-based (or shRNA-based) based loss-of-function studies would be needed.

## STAR★METHODS

### RESOURCE AVAILABILITY

#### Lead contact

Further information and requests for resources and reagents should be directed to and will be fulfilled by the lead contact, Jogender Tushir-Singh (jtsingh@ucdavis.edu).

#### Materials availability

All unique/stable reagents generated in this study are available from the lead contact with a completed materials transfer agreement. For further information, contact the lead contact.

#### Data and code availability

Data reported in this paper will be shared by the lead contact upon request as long as the requests do not infringe on our intellectual proprietary. This paper does not report the original code. Any additional information required to reanalyze the data reported in this paper is available from the lead contact upon request.

### EXPERIMENTAL MODEL AND SUBJECT DETAILS

#### Mice strains

6 to 8 weeks-old (Age), 20-25-g (Weight), both male and female (Sex) mice were used for tumor xenografts generation, *in vivo* efficacy studies, imaging studies as described in the text (see Key Resource Table). Following mice stains were used: C56BL/6 (Jackson laboratories), Balb/C (Jackson laboratories), immunodeficient BALB/c derived athymic Nude Foxn1^nu^/Foxn1^+^ (Envigo) and NOD.Cg Prkdc^scid^ Il2rg^tm1Wjl^/SzJ also called NSG mice. All animal procedures were conducted under the accordance of University of Virginia Institutional Animal Care and Use Committee (IACUC) and (DoD ACURO) approved protocols and conformed to the relevant regulatory standards.

#### Cell lines

The cell lines were used in the study is provided in the Key Resource Table. All the cell lines were maintained in DMEM, MEM, RPMI-1640 or other required optimal medium supplemented with 10% heat-inactivated fetal bovine serum (FBS), 2mM glutamine, 100 U/ml penicillin, and 100 μg/ml streptomycin (complete medium) unless otherwise specified as described (Shivange et al., 2018). MC38 cells (provided by S. Ostrand-Rosenberg, University of Maryland) were cultured in DMEM supplemented with 10% (vol/vol) FCS and 1mM penicillin/streptomycin. Patient derived cells lines were maintained in 20% FBS and 100 mM sodium pyruvate in RPMI 1640 media supplemented with glutamax (GIBCO) and 1% penicillin/streptomycin (GIBCO). Various cell lines were trypsinized and expanded as follow: After digestion, the cell suspension was neutralized with complete media and centrifuged 5 min at 1500 rpm. The cell pellets were suspended in relevant DMEM/RPMI media and either expanded or seeded after counting using countess II (Life technologies). Passaged cell lines were routinely tested for mycoplasma using MycoAlert Detection Kit (Lonza).

#### Pre-clinical and clinical DR5 antibodies

The sequences of various DR5 antibodies used in this manuscript is provided in the Table S1.

### METHOD DETAILS

#### Recombinant antibody Cloning

The sequence of various clinical DR5 agonist antibodies and recombinant DR5 proteins generated are provided in Tables S1 and S2. All antibodies were cloned and expressed as described earlier (Shivange et al., 2018). Wherever indicated bispecific antibodies were engineered by genetically linking CH3 Fc to lexatumumab, KMTR2, AMG655, tigatuzumab, MD5-1, etc. using flexible linker as described earlier (Shivange et al., 2018). The DNA sequences were retrieved from the open sources (Imgt.org or publicly available patents or rscb.org, PDB database) and synthesized as gene string using Invitrogen GeneArt gene synthesis services. After PCR amplification, DNA was gel purified and inserted into pCDNA 3.1 vectors (CMV promoter) by making use of In-Fusion HD Cloning Kits (Takara Bio). EcoR1 and HindIII digested vector was incubated with overlapping PCR fragments (of various recombinant DNAs, see list of clones in Key resources table) with infusion enzyme (1:2, vector: insert ratio) at 55°C for 30 min, followed by additional 30 min incubation on ice after adding *E. coli* Stellar™ cells (Clontech, see reagent Table). Transformation and bacterial screening were carried out using standard cloning methods. Positive clones were sequenced confirmed in a 3-tier method. Confirmed bacterial colonies were Sanger sequencing upon PCR followed by re-sequencing of mini-prep DNA extracted from the positive colonies. Finally, maxipreps were re-sequenced prior to each transfection. Recombinant antibodies were also re-confirmed by ELISA and flow cytometry surface binding studies as described earlier (Shivange et al., 2018).

#### Recombinant antibody expression

Various recombinant antibodies used in this study and recombinant target antigens were engineered, expressed, and purified in Singh Laboratory of Novel Biologics as described earlier (Shivange et al., 2018). Briefly, FreeStyle CHO-S cells (Invitrogen, Reagents Table) were cultured and maintained according to supplier’s recommendations (Life technologies) biologics using FreeStyle CHO expression system (Life technologies) and as previously described (Durocher and Butler, 2009; Shivange et al., 2018). A ratio of 1:2 (light chain, VL: heavy chain, VH) DNA was transfected using 1 μg/ml polyethyleneimine (PEI, see Key resources table). After transfections, cells were kept at 37°C for 24 hr. After 24 hr, transfected cells were shifted to 32°C to slow down the growth for 9 additional days. Cells were routinely fed (every 2^nd^ day) with 1:1 ratio of Tryptone feed and CHO Feed B (see Reagents Table). After 10 days, supernatant from cultures was harvested and antibodies were purified using protein-A affinity columns. Recombinant human Apo2L/TRAIL was obtained from R&D systems. His-tag Apo2L was also expressed and purified using nickel NTA columns (see Key resources table) using a standard BL21 bacterial expression system as described earlier (Shivange et al., 2018). His-Apo2L generated in our laboratory was confirmed (alongside commercial Apo2L) using multiple cancer lines. Similarly, the activity of commercial MD5-1 antibody was compared next to recombinant MD5-1 generated in our laboratory using various cancer cell lines as described earlier (Shivange et al., 2018).

#### Antibody purification

Various recombinant antibodies used in this study and recombinant target antigens were affinity purified using HiTrap MabSelect SuRe (GE, 11003493) columns in Singh Laboratory of Novel Biologics as described earlier (Shivange et al., 2018). Transfected cultures were harvested after 10 days and filtered through 0.2-micron PES membrane filters (Millipore Express Plus). Cleaning-in-place (CIP) was performed for each column using 0.2M NaOH wash (20 min). Following cleaning, columns were washed 3-times with Binding buffer (20 mM sodium phosphate, 0.15 M NaCl, pH 7.2). Filtered supernatant containing recombinant antibodies or antigens were passed through the columns at 4°C. Prior to elution in 0.1M sodium citrate, pH 3.0–3.6, the columns were washed 3 times with binding buffer (pH 7.0). The pH of eluted antibodies was immediately neutralized using sodium acetate (3M, pH 9.0). After protein measurements at 280 nm, antibodies were dialyzed in PBS using Slide-A-Lyzer 3.5K (Thermo Scientific, 66330). Antibodies were run on gel filtration columns (next section) to analyze the percent monomers. Whenever necessary, a second step size exclusion chromatography (SEC) was performed. Recombinants IgG4-Fc tagged extracellular domains of various rDR5 mutants, etc. were also similarly harvested and purified using protein-A columns.

#### Size Exclusion chromatography

The percent monomer of purified antibodies was determined by size exclusion chromatography. 0.1mg of the purified antibody was injected into the AKTA protein purification system (GE Healthcare Life Sciences), and protein fractions were separated using a Superdex 200 10/300 column (GE Healthcare Life Sciences) with 50mM Tris (pH 7.5) and 150mM NaCl. The elution profile was exported as an excel file and a chromatogram was developed. The protein sizes were determined by comparing the elution profile with the gel filtration standard (BioRad 151-1901) (Hong et al., 2012). Any protein peak observed in the void fraction was considered as antibody aggregate. The area under the curve was calculated for each peak, and a relative percent monomer fraction was determined as described earlier (Shivange et al., 2018)

#### Binding studies by ELISA

Binding specificity and affinity of various described IgG1 subclasses were determined by ELISA using the recombinant extracellular domain of DR5/TRAIL-R2. For coating 96-well ELISA plates (Olympus), the protein solutions (2 μg/ml) were prepared in coating buffer (100mM Sodium Bicarbonate pH 9.2) and 100 μL was distributed in each well. The plates were then incubated overnight at 4°C. The next day, the unbound areas were blocked by cell culture media containing 10% FBS, 1% BSA and 0.5% sodium azide for 2 hr at room temperature. The serial dilutions of antibodies (2-fold dilution from 50 nM to 0.048 nM) were prepared in blocking solution and incubated in target protein-coated plates for 1 hr at 37°C. After washing with PBS solution containing 0.1% Tween20, the plates were incubated for 1 hr with horseradish peroxidase-(HRP) conjugated anti-human IgG1 (Thermo Scientific, A10648). Detection was performed using a two-component peroxidase substrate kit (BD biosciences), and the reaction was stopped with the addition of 2N Sulfuric acid. Absorbance at 450 nm was immediately recorded using a Synergy Spectrophotometer (BioTech), and background absorbance from negative control samples was subtracted. The antibody affinities (Kd) were calculated by non-linear regression analysis using GraphPad Prism software.

#### *In vitro* cell viability Assays

Cell viability following Apo2L, lexatumumab, KMTR2, tigatuzumab, AMG-655, MD5-1, 2DEI, etc. treatments (as indicated in various figures) either alone or in combination with an anti-Fc reagent were determined using the AlamarBlue cell viability assays and MTT cell proliferation assays as per manufactured protocols. Briefly, cells (indicated cells in the main text or figure legends) were treated with increasing concentrations of various antibodies (as indicated) along with relevant positive and negative control antibodies for 6 hr, 24 hr, or 48 hr (as indicated according to the experiment). For each cell killing assay, the figures show the representative profiles from n = 2-4 with different cultured confluency. Whenever used for immunoblotting, following antibodies treatment caspase-3 processing in tumor cells was monitored using selective antibodies that recognize cleaved human caspase-3 or total caspase-3 (Cell signaling, 9661 and 9668). TRAIL-R2 receptor in oligomerization was determined using immunoblotting assays (cell signaling Rabbit mAb, 8074). Cell viability was additionally examined by flow cytometry-based apoptotic detection methods using 7-ADD exclusion from live cells as described earlier (Shivange et al., 2018).

#### IC_50_ Determination

IC_50_ values were calculated using MTT assays. Cells were seeded in 96 wells plates. Next day, when cultures became adherent, cells were incubated for 48 hr at 37°C (5% CO2) with the increasing concentrations of the antibodies or drug (such as cisplatin) as indicated in experiments. Before treatments, various antibodies were dialyzed into PBS and typically had a pH of around 7.5. Values obtained after reading the 96 well plates were normalized to IgG control antibody control, and IC_50_ values were calculated using GraphPad Prism software using nonlinear dose-response regression curve fits. The final results shown in the histograms were obtained from three independent experiments. Whenever provided in the turns, the error bars show ± SEM.

#### Western Blotting

Cells were cultured overnight in tissue culture-treated 6-well plates before treatment. After antibody treatment for the indicated time, cells were rinsed with PBS and then lysed with RIPA buffer supplemented with protease inhibitor cocktail (Thermo Scientific). Spinning at 14000 rpm for 30 min cleared lysates, and protein was quantified by Pierce BCA protein assay kit. Western blotting was performed using the Bio-Rad SDS-PAGE Gel system. Briefly, 30 μg of protein was resolved on 10% Bis-Tris gels and then transferred onto PVDF membrane. Membranes were blocked for one hour at room temperature in TBS + 0.1% Tween (TBST) with 5% non-fat dry milk. Membranes were probed overnight at 4°C with primary antibodies. Membranes were washed three times in TBST and then incubated with anti-rabbit or anti-mouse secondary antibodies (1/10,000 dilution, coupled to peroxidase) for 1 hr at room temperature. Membranes were then washed three times with TBST, and Immunocomplexes were detected with SuperSignal West Pico Chemiluminescent Substrate (Thermo Fisher Scientific). Images were taken using a Bio-Rad Gel Doc Imager system. Primary antibodies are listed in the Key resources table.

#### Pre-neutralization assays

Whenever indicated throughout the manuscript text or in figure legends, variable domain pre-neutralization of DR5 agonist antibodies was carried out. For *in vitro* and *in vivo* studies indicated antibodies and indicated recombinant antigens (rDR5 etc.) were incubated together (either 1:1 or indicated ratio) at 37°C for 1 hr shaking on a platform. As a control, indicated non-neutralized antibodies were also incubated at 37°C for 1 hr shaking on a platform either with PBS alone or with recombinant non-specific proteins such as rHER2 or rGFP. Following pre-neutralization, antibodies were either used *in vitro* for cell killing assays, or for cellular/tumor lysates generation (immunoblotting), or for *in vivo* imaging, etc. as indicated.

#### Flow cytometry

The cell surface expression of huDR5, muDR5, DR5 (L), DR5 (3RE), DR5 (3RA), etc. and other surface proteins were analyzed by flow cytometry. Overnight grown cells were trypsinized and suspended in FACS buffer (PBS containing 2% FBS). The single-cell suspension was then incubated with primary indicated antibodies for 1 hr at 4°C with gentle mixing. Wherever indicated PFA was using for cross-linking the antibodies. Following wash with FACS buffer, the cells were then incubated with the fluorescently labeled secondary antibody for 1 hr. Cells were washed and flow cytometry was performed using FACSCalibur. The data was analyzed by FCS Express (*De Novo* Software) and FlowJo.

#### Mechanical dissociation of tumors to obtain single-cell suspensions

Viable single cells from tumor tissues were isolated as described here (Leelatian et al., 2017). Briefly, after indicated antibody treatments (4-6 doses), mice were euthanized, and tumors were harvested using sterile scissors and forceps. After the tumor excision, tissues were minced into small pieces in sterile RPMI-1640 media using two single-edged razor blades. Small tumor pieces were passed through a 70 μm cell strainer in sterile RPMI-1640 media. A rubber plunger and syringe were used to mesh the dissociated cells through the cell strainer, and media containing dissociated cells was collected onto a sterile labeled conical tube. Dissociated tumor cells were subjected to flow cytometry (FACS) expression as described under flow cytometry protocol.

#### Native DR5 immunoprecipitation studies with clinical antibodies

Native DR5 immunoprecipitation studies with clinical antibodies were performed as described earlier (Mondal et al., 2021). Briefly, cells were cultured in 10 cm tissue culture dishes for 24 hours prior to treatment. Before treatment, the culture medium was replaced with a serum-free medium. Cells were treated with indicated DR5 agonists (IgG1-Fc) antibodies (10, 50 or 500nM) for an indicated time as described in the text and figures. Cells were harvested and lysed with IP lysis buffer (20mM Tris pH 7.5, 150mM NaCl, 1mM EDTA, 10% Glycerol, 1% Triton-X, 0.5mM PMSF) supplemented with protease inhibitor cocktail (Thermo Scientific). Spinning at 14000 rpm for 30 min collected clear protein lysates, and protein was quantified by Pierce BCA protein assay kit. 1-1.5 mg protein (~400-500μl) was taken into the Eppendorf microcentrifuge tube. Protein lysates were incubated with anti-human IgG1-Fc specific beads for 2 hr at 4°C, placing them into a rotating wheel. Protein conjugated beads were washed thrice with phosphate buffer saline (PBS). Finally, beads were boiled at 100°C for 5 minutes with 30μl of SDS sample buffer. The 15-20μl sample was loaded into the SDS-gel, and western blotting was performed using the Bio-Rad SDS-PAGE Gel system followed by immunoblotting using indicated DR5, caspase-8, etc. specific antibodies.

#### Generation of CRISPR DR5 Knockout tumor cells

Using Synthego’s Gene Knockout Kit v2, we performed genomic deletion of DR5 in various tumor cell lines. Briefly, per manufacturer’s instructions, ribonucleoprotein (RNP) complexes consist ingof purified Cas9 nuclease duplexed with chemically modified synthetic guide RNA (sgRNA) targeting DR5 were delivered to the cell lines using Lipofectamine CRISPRMAX Transfection Reagent. DR5 knockout lines were further selected by treatment with a 200nM DR5 agonist antibody, which initiates apoptosis in leftover wild-types cells to get a complete DR5knockout population. In detail, we first prepared plates by pre-warming 2 × 24-well cell culture plates with 500 μL of standard growth medium in each well. Next, RNP complexes were assembled in a 1.3:1 sgRNA to Cas9 ratio and working concentrations (3 pmol/μl) of RNPs were prepared in a microcentrifuge tube (Tube 1). Next, transfection solution was prepared in a separate microcentrifuge tube (Tube 2) that contained Lipofectamine CRISPRMAX reagent in Opti-MEM I reduced serum medium. The concentrations were used in a 26.5 mL reaction volume as per manufacture protocol. The transfection solution (Tube 2) was directly added to the RNPs mix (Tube 1) and mixed well by pipetting up and down. The mix (~50 μl) was next incubated for 10 minutes at room temperature followed by addition on freshly trypsinized cells in growing phase 0.42 – 1.2 × 10^5^cells in 500 μL of the growth medium. The mix and cells with 500 μL growth media were distributed into two wells. Media was replaced after 24 hours of incubation and cells were allowed to grow for 2-3 days. The cells on the first plate were lysed and processed to analyze editing efficiency. The cells on the second plate were cultured for use in assays, banking, and/or single-cell cloning.

#### huDR5 (L), DR5 (3RE), DR5 (3RA) etc. stable transient line generation

Transfection of various DR5 constructs into the different tumor cell lines was achieved by jetOPTIMUS DNA transfection reagent for recombinant DR5 cloned in pCDN3.1 vector. In brief, 60%-70% of confluent cells were grown in a 10cm culture dish. Mixing 10ug of plasmid DNA and 10ul of transfection reagent into 1ml of jetOPTIMUS buffer made transfection solution. After incubating for 10min at room temperature, the transfection mix was added on the cells. The cells were further allowed to grow for 24 hours and then selected using 2 mg/ml of G418. In detail, 10 μg DNA was diluted into 1000 μL jetOPTIMUS buffer and vortexed. This was followed by the addition of 10 μL jetOPTIMUS into the DNA solution (ratio 1:1 corresponding to μg DNA: μL reagent) and vortexed and spun down briefly. The mixture was incubated for 10 minutes at room temperature. Next, the transfection mix was added dropwise onto the cells in a serum-containing medium and distributed evenly. Plates were incubated at 37°C for 24hrs. The next day transfection medium was replaced with by cell growth medium and cells were allowed to grow for another day before starting G418 (2 mg/ml) selection. Media was changed every day, and a reliable GFP signal was evident 72 hours of transfection. For long-term stable line generation lentiviral method was used as described in next section.

#### Lentiviral preparation and transduction

Lentiviral packaging and delivery were executed using the technology from system Biosciences, and the method was very similar to described here (Wollebo et al., 2013). Briefly, lentivirus was prepared by transfecting 293T cells in a T75 flask with transfer vector (6, μg) and packaging vectors (3 μg each) in the ratio of 2:1:1:1 using 30ug of PEI. The virus-containing culture medium was collected 48 and 72 h after transfection, cleared by filtration (0.45 μm Millipore, Bedford, MA) and concentrated by 20% PEG 6000. After centrifugation at 3000 g for 30 mins, the pellet was resuspended in 1/10^th^ of the initial volume in phosphate-buffered saline (PBS)/0.1% bovine serum albumin (BSA), stored at −70°C. For transduction, the 60%-70% confluent cells were plated in a 10 cm plate, and 5ml virus along with 5ug/ml polybrene was added. Transduction Medium was replaced with growth medium after 12hour and allowed the cells to grow for another 24 hours. The transduced DR5 positive cells were selected using 2.5 μg/ml puromycin. In detail, HEK293T cells were cultured in high glucose-containing Dulbecco’s modified Eagle’s medium (DMEM; Corning) supplemented with 10% fetal bovine serum (FBS, Corning) at 37°C at 5% CO2. For transfection, the cells were seeded a day before at a density of 70%–80% in 10 cm culture dish. The transfection mix was prepared as following: Transfer plasmid with gene of interest (6 μg), pVSVG plasmid (3 μg), pREV (3 μg), pRRE (3 μg), OptiMEM media (500 μl) and PEI (30 μg). Transfection mixture was vortex mixed and quick centrifuged, and incubated in room temperature for 10min. Transfection mixture was then added gently on the cells through the wall and mix by tilting the plate. Transfected cells were incubated at 37°C for 12-16 hr, and the medium was replaced with 10ml of fresh growth medium. Virus containing culture media was collected after 48 hr and 72 hr of incubation. The floating cell debris was separated by quick spin at 1000rpm for 5 min and then virus-containing culture medium was filtered through 0.45 μm Millipore, Bedford, MA. Next, the virus was concentrated by using PEGylation in the following ratio: Virus suspension (40 ml), 50% PEG 6000 (10 ml) and 5M NaCl (1 ml). PEGylated solution was mixed and incubated at 4°C overnight on a gentle rocker. The precipitated virus particles were centrifuged at 4000rpm for 30min and resuspended in 4ml of culture medium. To transduce, the tumors cells were seeded a day before at a density of 60%-70% in a 10 cm culture plate. Transduction solution was prepared by mixing 2ml of the virus, 5 μg/ml of polybrene and 1x HEPES buffer and gently added on to the cells with 8ml of culture medium. Cells were allowed to grow at 37°C for additional 12 hr and then the medium was replaced with a growth medium. The 2.5 μg /ml puromycin selection was performed after 48 hr of transduction, and the medium was replaced each day with intermediate PBS washing to avoid the accumulation of dying cell debris. Stable cells appeared within a week.

#### Tumor xenograft studies

All animal procedures were conducted according to the University of Virginia Institutional Animal Care, and Use Committee (IACUC) and (DoD ACURO) approved protocols and conform to the relevant regulatory standards. Details of mice strains, age and sex used are provided above. Briefly, 6 to 8 weeks-old (Age), 20-25-g (Weight), both male and female (Sex) mice were used for tumor xenografts generation, *in vivo* efficacy studies, imaging studies, TIL isolation studies as described in the text. Following mice stains were used: C56BL/6 (Jackson laboratories), Balb/C (Jackson laboratories), immunodeficient BALB/c derived athymic Nude Foxn1^nu^/Foxn1^+^ (Envigo) and NOD.Cg Prkdc^scid^ Il2rg^tm1Wjl^/SzJ, also called NSG mice. Various different indicated solid cancer cell lines were used for tumor nude xenograft studies as described in the text. Weight and age (6-8 weeks old) matched mice were injected subcutaneously (SC) in their right flank or in breast fat pad or intraperitoneally with indicated cell lines in matrigel as indicated in various figures. Different cell number was injected as some cells were highly effective and some required higher density during xenografts. Tumor cells were mixed 100 μL volume with matrigel. For antitumor efficacy studies, mice bearing ~100 mm^3^ tumors were weight-matched. The animals were randomly assigned into groups and injected (25 μg, 50 μg, 100 μg, or indicated different doses) intraperitoneally as indicated in figure legends. The antibody doses were given two or three times per week as indicated in text and figure legends. Tumors were measured in two dimensions using a caliper as described previously (Graves et al., 2014; Mondal et al., 2021; Shivange et al., 2018; Wilson et al., 2011). Tumor volume was calculated using the formula: V = 0.5axb^2^, where a and b are the long and the short diameters of the tumor, respectively. (n = 4-6 animals were used for each therapeutic antibody injection). The p values are determined by a two-tailed paired Wilcoxon Mann-Whitney test (Takeda et al., 2008). For tumor regression studies, mice bearing ~100 mm^3^ tumors were (after matching tumor size, n = 4-7) randomly assigned into groups and injected with therapeutic antibodies as indicated (50-100 μg dose) intraperitoneally two-three times per week. Studies with UCD52 TNBC PDX tumors and WHIM-30, HCI-01 *in vitro* PDX cell lines were carried out by VCU PDX core and a per-diem fee was paid for this work. All DR5 agonist antibodies were engineered with IgG1 KO-Fc and S267E mutations. Tumors were measured two-three times a week and volumes were calculated as the product of three orthogonal diameters similar to nude animal studies as described in previous section. The p values are determined by a two-tailed paired Wilcoxon Mann-Whitney test. For Biochemical analysis, signal cell isolation or TIL isolations from tumors, mice were euthanized after indicated antibody treatment followed by tumor extraction (Li and Ravetch, 2012; Wilson et al., 2011).

#### Binding studies by BioLayer Interferometry (BLI)

Binding measurements were performed using Bio-Layer Interferometry on FortéBio Red Octet 96 instrument (Pall) as described earlier (Shivange et al., 2018). Biotin-Streptavidin-based sensors were employed for the studies. Recombinant IgG4-Fc linked DR5 variants were biotinylated using EZ-Link Sulfo-NHS-SS-Biotin (Thermo Scientific 21331) following the manufacturer’s instructions. Unreacted Sulfo-NHS-SS-Biotin reaction was quenched by 50mM Tris-Cl pH 7.4 and removed via dialyzing against PBS. For binding analysis, biotinylated antigens (1 μg/mL) were immobilized on streptavidin (SA) biosensors (Pall) for 300 s to ensure saturation. Associate and dissociation reactions were set in 96-well microplates filled with 200 μL of unbiotinylated DR5 agonist for 900 Sec. All interactions were conducted at 37°C in PBS buffer containing 2mg/ml BSA. These binding observations were also confirmed by biotinylated antibodies and probed against unbiotinylated DR5-Fc variants. Kinetic parameters (K_ON_ and K_OFF_) and affinities (KD) were analyzed using Octet data analysis software, version 9.0 (Pall).

#### DR5 lysate clustering assays

MDA-MB-436, OVCAR3, HCC1806, MDA-MB-231(WT), KO-MDA-MB-231-DR5 (L), KO-MDA-MB-231-DR5 (3RE), KO-MDA-MB-231-DR5 (3RA), etc. cells were cultured at 37°C, 5%CO_2_ in DMEM medium supplemented with 10% FBS. 0.3 X 10^6^ cells were seeded in each well of 6-well plates and grown at the above-mentioned conditions. When cells reached 80%–90% confluency, the experiment was started. First, the old media was removed followed by washing of cells with pH 8.0 PBS. Then, cells were incubated with DR5 agonist antibodies in pH 8.0 PBS for 1 hr at the 4°C with slow rotation. Just before use, 50mM BS3 solution was prepared (freshly) by dissolving 10 mg BS3 in 350 ml of water. 2 mM BS3 crosslinker working-solution was added to each well of 6-well plate. The plate was allowed for crosslinking reaction at room temperature for 45 minutes. After crosslinking, sample was quench and unreacted BS3 with 40 mM Tris-HCl pH 7.5 at room temperature for 10-15 minutes. Finally, cell lysate was prepared in RIPA buffer and immunoblotting was carried out in non-reducing denaturing conditions.

#### Myocardial Experimental metastatic TNBC model for metastatic *in vivo* efficacy studies

All animal studies performed were in accordance with institutional guidelines at the Animal Care and Use Committee and have been described earlier by our group (Przanowski et al., 2020). A close-chested method was used to inject cells in the left ventricle of mice’s hearts to establish a metastatic mice model. NSG (NOD SCID gamma) female mouse was anaesthetized with 2.5% isoflurane (97.5% oxygen), then 30 G needle was inserted into the left ventricle of the mouse to inject 100 μl of PBS containing 0.3 million MDA-MB-231-2B cells. Antiseptic wipes were used both before and after injection to prevent infection. Sterile eye ointment was applied in order to prevent corneal drying and a heat pad was used to maintain body temperature. The proliferation and localization of cells were monitored by bioluminescence at UVA-Molecular Imaging Core (IVIS-Spectrum). Antibody treatment was started after 2-3 weeks of injections as indicated in figure legends. Each indicated antibody was injected intraperitoneally 100μg/mice, 2 times a week as indicated.

#### Recurrent TNBC tumor model by surgical resection of primary tumor

All animal studies performed were in accordance with institutional guidelines at the Animal Care and Use Committee. For the resection experiment, first the primary tumor was formed with 5X10^6^ MDA-MB-231-Luc-D3H2LN-BMD2b (provided by Takahiro Ochiya) cells subcutaneously injected in the right flank of 7-8-week-old female NSG (NOD SCID gamma) mice and allowed for tumor growth. Primary tumor growth was evaluated using calipers every two days and every day once it reached 1cm. Tumors were measured in two perpendicular dimensions, and the volume was estimated using the formula [volume = 0.52 x(width)^2^ x (length)] for approximating the volume (mm^3^) of an ellipsoid. Tumor excision was performed under anesthesia when tumor size reaches 700-800 mm^3^ in size. Mice were treated with Buprenorphine (0.05 mg/kg) 1 hr before surgery for pain management, and the tumor resection was performed under anesthesia using 2.5% isoflurane. Surgeries were performed with sterile tools on a sterile surface in a sterile hood. Hairs were removed from the surgical site one day prior. Betadine was applied to the surgery site with clean gauze in a circular fashion, starting at the surgical incision site and rotating outward. Then 70% alcohol was applied. The procedure was repeated three times, discarding the cotton pad after each use. After tumor resection, the resulting wound was closed with sterile sutures. Sterile eye ointment was applied to prevent corneal drying, and a heating pad was used to maintain body temperature. Animals were returned to their cages after they were fully alert and demonstrating no complications from the procedure. Post-surgery animals were monitored hourly for 24 hr period. The number and placement of metastasis were measured by bioluminescence at UVA-Molecular Imaging Core (IVIS-Spectrum) every week for the initial three weeks, then thrice in a week as indicated in text and figure legends. Antibody treatment was started after a week of tumor resection. Each indicated antibody was injected intraperitoneally 100μg/mice every 3rd day or 2 times a week average.

### QUANTIFICATION AND STATISTICAL ANALYSIS

Data, unless indicated otherwise, are presented as mean ± SEM. In general, when technical replicates were shown for *in vitro* experiments, a Student’s t test was used for statistical analysis, and the same experiment was at least repeated once with a similar trend observed. When data from multiple experiments were merged into one figure, statistical significance was determined by a Wilcoxon Mann-Whitney test using Graph Pad Prism 5.0 software.Tumor growth curves are displayed as mean ± SEM. For all the statistical experiments, the p values, p < 0.05 (*), p < 0.01 (**) and p < 0.001 (***) were considered statistically different or specific p values indicated otherwise or “ns” indicates non-significant.

## Supplementary Material

1

## Figures and Tables

**Table:** KEY RESOURCES TABLE

REAGENT or RESOURCE	SOURCE	IDENTIFIER
Antibodies		
Caspase-8	Cell Signaling Technology	Cat #9746
Folate Receptor alpha Polyclonal Antibody (FOLR1)	Invitrogen	Cat # PA5-24186
FOLR1	R & D System	RRID:AB_2278620
Cleaved PARP antibody	Cell Signaling Technology	RRID:AB_2160739
Anti-Rabbit-HRP antibody	Cell Signaling Technology	RRID:AB_2099233
Anti-Mouse-HRP antibody	Cell Signaling Technology	RRID:AB_330924
Commercial MD5-1 (anti-Murine DR5) antibody	Abcam	RRID:AB_10687929
Cy5 conjugated Anti-Human IgG (H+L) Alexa Fluor® 488 AffiniPure Goat Anti-Rabbit IgG (H+L)	Jackson ImmunoResearch	RRID:AB_2340539
Cy5 AffiniPure Donkey Anti-Mouse IgG (H+L) Anti-Human IgG1 HRP	Jackson ImmunoResearch	RRID:AB_2340819
Anti-DR5 (Human)	Life Technologies	RRID:AB_468592
Anti-DR5 (Human)	Cell Signaling Technology	RRID:AB_10692107
Anti-DR5 (Human)	Abcam	Cat #ab199357
Anti-DR4 (Human)	Cell Signaling Technology	RRID:AB_2799223
Cleaved Caspase-3 Antibody	Cell Signaling Technology	RRID:AB_2341188
Capase-3 Antibody	Cell Signaling Technology	RRID:AB_2069870
GAPDH Antibody	Cell Signaling Technology	RRID:AB_10622025
Goat anti-Human IgG (Fc) PE	Thermo Fisher	RRID:AB_465926
Goat anti-Human IgG (Fc) magnetic	Polysciences	Cat # 84324-50
Bacterial and virus strains		
*E. coli* HST08, Stellar Cells	Takara BioScience	Cat # 636766
Biological samples (antibodies)		
Lexatumumab	Imgt.org	See Table S1
AMG655	Imgt.org	See Table S1
Apomab	Rscb.org	See Table S1
Tigatuzumab	Imgt.org	See Table S1
KMTR2	Rscb.org	See Table S1
Chemicals, peptides, and recombinant proteins		
WT DR5 recombinant protein	NCBI: NP_003833.4	See Table S2
3RA DR5 recombinant protein	NCBI: NP_003833.4	See Table S2
3RE DR5 recombinant protein	NCBI: NP_003833.4	See Table S2
Critical commercial assay reagents and kits		
HiPure Plasmid Maxiprep kit	Invitrogen	Cat # K21007
Infusion	Takara BioScience	STO344
Cell proliferation Assay	TACS MTT Trevigen	Cat# 4890-25-K
HiTrap MabSelect SuRe	GE Healthcare	Cat # 11-0034-93
TMB Substrate Reagent Set	BD OptEIA	Cat # 555214
CHO free style Media	Thermo Fisher	Cat # 12651014
HiTrap MabSelect Sure column	GE	Cat # 11003493
Protein-A resin	Thermo Fisher	Cat # P153142
CHO CD efficient Feed B	Life Technologies	Cat # A1024001
Matrigel	Corning	Cat # 354234
Corning® 500 mL RPMI 1640	Corning	Cat # 10-040-CV
Corning® 500 mL DMEM (Dulbecco’s Modified Eagle’s Medium)	Corning	Cat # 10-13-CV
Halt protease inhibitor	Thermo Fisher	Cat # 78430
Goat anti-Human IgG (H&L) Coated Magnetic Particles, Smooth Surface	Spherotech	Cat # HMS-30-10
Cy5-NHS Ester	Gold Biotechnology	Cat # B-430-1
Infusion	Takara BioScience	Cat # 638920
CHO CD efficient Feed B	Life Technologies	Cat # A1024001
PEI transfection reagent	Thermo Fisher	Cat # BMS1003A
Stellar Cells	Takara BioScience	Cat # 636766
BLI Biosensor	ForteBio	Cat # 18-5019
Xenolight D-luciferin potassium salt	PerkinElmer	Cat # P/N 122799
PEI transfection reagent	Thermo Fisher	Cat # BMS1003A
HisPur Ni-NTA resin	Thermo Fisher	Cat # 88221
EZ-Link Sulfo-NHS-SS-Biotin	Thermo Fisher	Cat # 21331
Experimental models: Cell lines		
Human: OVCAR-3	Ovarian Cancer	ATCC HTB-161
Human: MDA-MB-436	TNBC	ATCC HTB-130
Human: MDA-MB-231	TNBC	ATCC HTB-26
Human: MDA-MB-231-2B	TNBC	ATCC HTB-26
Human: A549	Lung Cancer	ATCC CLL-185
Human: Cavo-3	Ovarian Cancer	ATCC HTB-75
Human: HCC1806	TNBC	ATCC CRL-2335
Mouse: 4T1	Murine TNBC cell, Gift from Kevin Janes, UVA,	ATCC CRL-2539
Mouse: MC38	Murine Colon Cancer, Gift from Dr. Suzanne Ostrand-Rosenberg, UMBC	CVCL_B288
Mouse: 4T1 Chimeric human-mouse DR5 no G4S linker	Generated in our laboratory (https://doi.org/10.15252/emmm.202012716)	Human DR5 expressing murine TNBC cells
Human: MDA-MB-231 DR5-KO	Generated in our laboratory (This paper)	DR5 knockout cells
Human: MDA-MB-231 DR5-KO-WT DR5	Generated in our laboratory (This paper)	WT DR5 expressing cells
Human: MDA-MB-231 DR5-KO and 3RE mutant DR5	Generated in our laboratory (This paper)	3RE mutant DR5 expressing cells
Human: MDA-MB-231 DR5-KO and 3RA mutant DR5	Generated in our laboratory (This paper)	3RA mutant DR5 expressing cells
Experimental models: Mouse		
Mouse: Athymic Nude Foxn1nu	Envigo	Order code 069
Mouse: C56BL/6	Jackson Lab	000664
Mouse: NOD.Cg-*Prkdc^scid^ Il2rg^tm1Wjl^*/SzJ	Jackson Lab	005557
Mouse: BALB/c	Jackson Lab	000651
Software and algorithms		
Vector NTI	Thermo scientific	N/A
GraphPad Prism	GraphPad Software	https://www.graphpad.com:443/
FlowJo	FlowJo, LLC	https://www.flowjo.com/
FCS Express	De Novo Software	https://www.denovosoftware.com/
Chimera	UCSF Chimera	https://www.cgl.ucsf.edu/chimera/
Other		
ChemiDoc imaging system	Bio-Rad	N/A
AKTA pure chromatography system	GE Healthcare	N/A
Bio Rad BioLogic LP chromatography system	Bio-Rad	N/A
Minitron Incubator Shaker	INFORS HT	N/A
Cytiva HiTrap Column	Cytiva/GE Healthcare	N/A

## Highlights

- Identification of DR5 negative regulatory patch of positively charged residues (PPCR)
- PPCR-targeting antibodies maintains unexpectedly low DR5 surface binding
- PPCR potentially contributes to ectodomain inhibition in the absence of DR5 agonists
- Characterization of a highly superior PPCR dual-specificity strategy co-targeting DR5
