# Supplementary material for: A patch of positively charged residues regulates the efficacy of clinical DR5 antibodies in solid tumors
Source: Cell Rep. Author manuscript; Available in PMC 2022 Jan 1. (PMC8720280; doi:10.1016/j.celrep.2021.109953)
Supplement: 1 [file NIHMS1753650-supplement-1.pdf]

Cell Reports, Volume *37*

Supplemental information

A patch of positively charged residues regulates the efficacy of clinical DR5 antibodies in solid tumors

Gururaj Shivange, Tanmoy Mondal, Evan Lyerly, Sanchita Bhatnagar, Charles N. Landen, Shivani Reddy, Jonathan Kim, Britney Doan, Paula Riddle, and Jogender Tushir-Singh

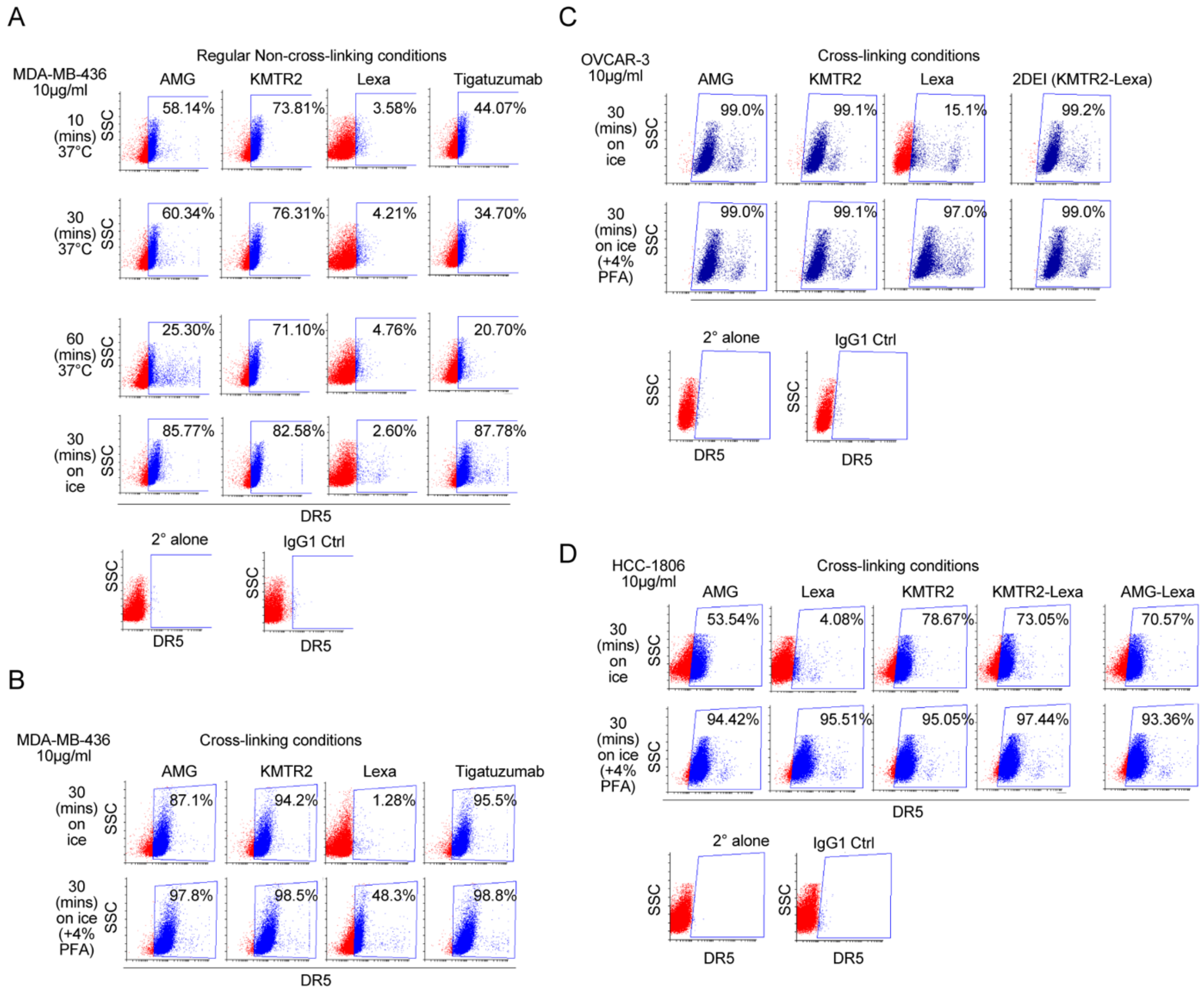

Figure S1. Tumor cell surface binding flow cytometry analysis of various DR5 agonists, Related to Figure 1

(A-D) Indicated tumor cells were examined using flow cytometry by using indicated DR5 agonist antibodies (IgG1-Fc) after treatments for the indicated times. DR5 agonist antibodies were added to the cells under indicated conditions such as room temperature, ice, or ice combined with a crosslinking reagent. Secondary anti-IgG1-Fc and IgG1 antibody controls were included for all the cell lines.

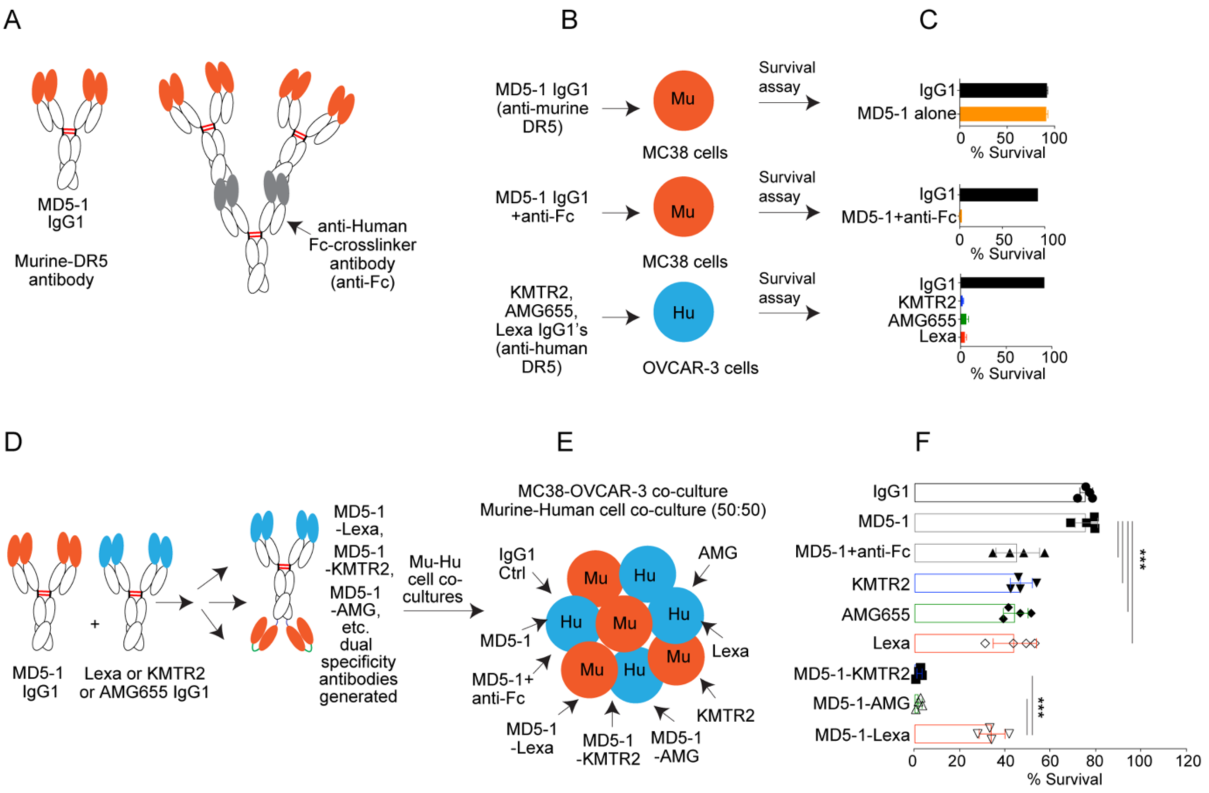

Figure S2. Lexatumumab is not an effective surface crosslinker for murine DR5 antibody, Related to Figure 1

(A) Schematic showing murine DR5-agonist MD5-1 working mechanism via anti-Fc cross-linking.

(B-C) Comparison of % cell viability by human DR5 agonists and MD5-1± anti-Fc cross-linking secondary antibody.

(D) Genetic construction details of the generation of MD5-1 containing indicated bispecific antibodies.

(E) A human-murine tumor cell co-culture schematic showing treatment with indicated antibodies followed by cell viability assays.

(F) Cell viability assays of indicated human-DR5 agonists, MD5-1 and various bispecific antibodies in the co-culture antibodies (n=3).

Error bars in (F) represent SD (n=3).

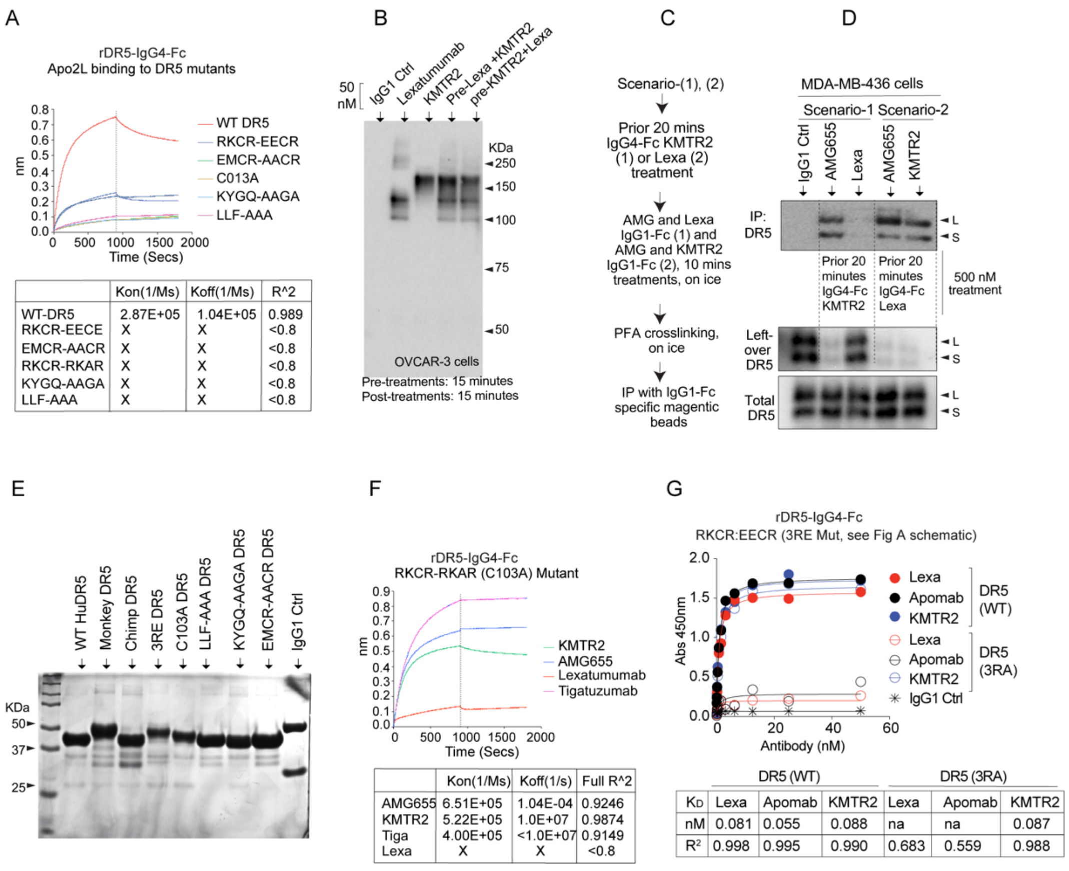

Figure S3. *In-vitro* binding and surface pulldown analysis of Apo2L and DR5 agonists, Related to Figure 2 and 3

(A) The binding kinetics of immobilized biotinylated rDR5 (having indicated mutations) against Apo2L was measured using BLI. All indicated mutations interfered with Apo2L binding.

(B) OVCAR-3 tumor cells were treated with IgG1, lexatumumab (50nM), KMTR2 (50nM) and lexatumumab+KMTR2 (50nM) for 20 minutes. After indicated antibody treatments (top), cellular lysates were analyzed in non-reducing denaturing gels and immunoblotting with anti-DR5 antibody (See methods).

(C-D) Scenario-1: MDA-MB-436 tumor cells were pre-treated with KMTR2 IgG4-Fc for 20 minutes, followed by treatment with Lexa IgG1 or AMG655 IgG1 (additional 10 minutes) on ice in the presence of crosslinking solution. Scenario-2: MDA-MB-436 tumor cells were pre-treated with lexatumumab IgG4-Fc for 20 minutes, followed treatment with KMTR2 IgG1 or AMG655 IgG1 (additional 10 minutes) on ice in the presence of crosslinking solution. Immunoprecipitation was carried out using anti-IgG1-Fc specific beads followed by immunoblotting using DR5 specific antibodies. Unbound supernatant and total lysates were also analyzed from the same experimental conditions using immunoblotting.

(E) Various indicated recombinant IgG4-Fc tagged DR5 proteins were run on SDS-PAGE in reducing conditions. IgG1 serves as a control for size.

(F) The binding kinetics of immobilized biotinylated C103A rDR5-IgG4-Fc (RKCR-RKCR) against AMG655-IgG1, lexatumumab-IgG1, KMTR2-IgG1, and tigatuzumab IgG1 were measured.

(G) Binding affinity comparison of KMTR2, Lexa, and apomab IgG1 antibodies were determined by ELISA using either WT DR5 or 3RE-DR5.

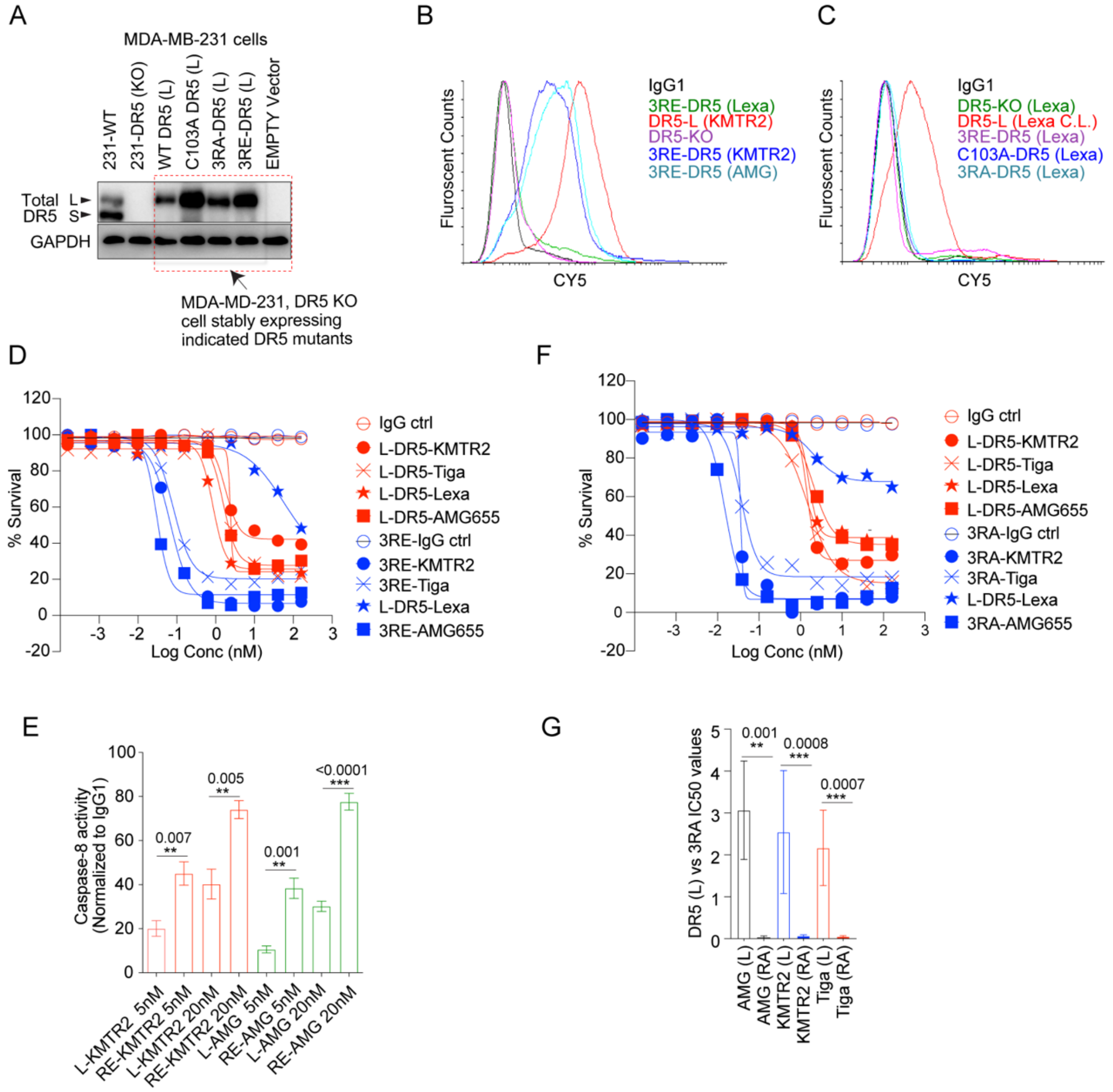

Figure S4. PPCR substitution increases cytotoxic activity of DR5 agonists, Related to Figure 4

(A) DR5 knock-out MDA-MB-231 cells were stably transfected with lentiviruses expressing indicated mutations in human DR5. After selection, total cellular lysates were run on a gel and immunoblotted using a commercial anti-DR5 antibody. Lane 1 has the whole cell lysates from WT MDA-MB-231 cells showing both DR5(L) and DR5(S).

(B-C) DR5 WT(L), DR5 3RE(L), DR5 3RA(L) expressing MDA-MB-231 cells were incubated with KMTR2 and lexatumumab on ice in the presence of PFA, followed by flow cytometry analysis.

(D) Dose-dependent cell killing of DR5 knock-out MDA-MB-231 cells stably expressing either DR5 WT(L) and DR5 3RE(L) in the presence of indicated DR5 agonist antibodies.

(E) Caspase-8 assay was carried out as per manufacture instructions using DR5 WT(L) and DR5 3RE(L) expressing MDA-MB-231 cells at indicated concentrations of DR5 antibodies.

(F) Dose-dependent cell killing of DR5 knock-out MDA-MB-231 cells stably expressing either DR5 WT(L) and DR5 3RA(L) in presence of indicated DR5 agonist antibodies.

(G) Fold change in IC50 values from L (n=3).

Error bars in (E) and (G) represent SEM and SD respectively (n=3).

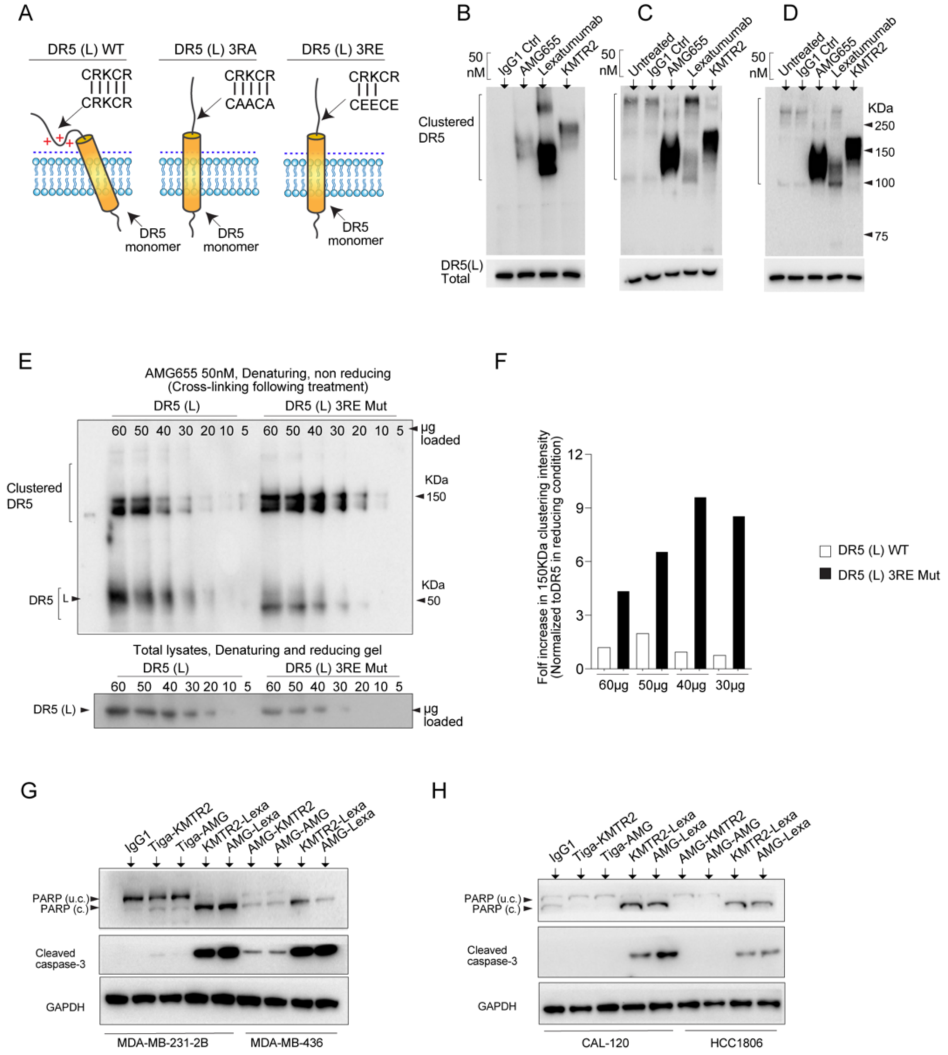

Figure S5, PPCR substitution increases clustering function of DR5 agonists, Related to Figure 4 and 5

(A) Cartoon schematic showing potential ECD autoinhibition due to WT-DR5(L) but not DR5-3RE or DR5-3RA.

(B-D) WT-DR5, DR5-3RE, and DR5-3RA stable MDA-MB-231 cells were analyzed for DR5 clustering in non-reducing denaturing gels after indicated antibody treatment. Total DR5 was immunoblotting in reducing conditions as a loading control.

(E-F) WT-DR5(L), DR5-3RE mutant stable MDA-MB-231 (expressing an equal level of surface DR5) were treated with AMG655 antibody. Quantitated cell lysates with equal protein concentrations (from two different lines) were loaded on the same gel and analyzed for clustered DR5 using immunoblotting. Normalized quantitation of clustered DR5 signal intensity from G confirms significantly high DR5 clustering in 3RE lysates.

(G-H) Immunoblotting of PARP and caspase-3 from indicated cell lysates after indicated 2DEI and random bispecific antibody treatments.

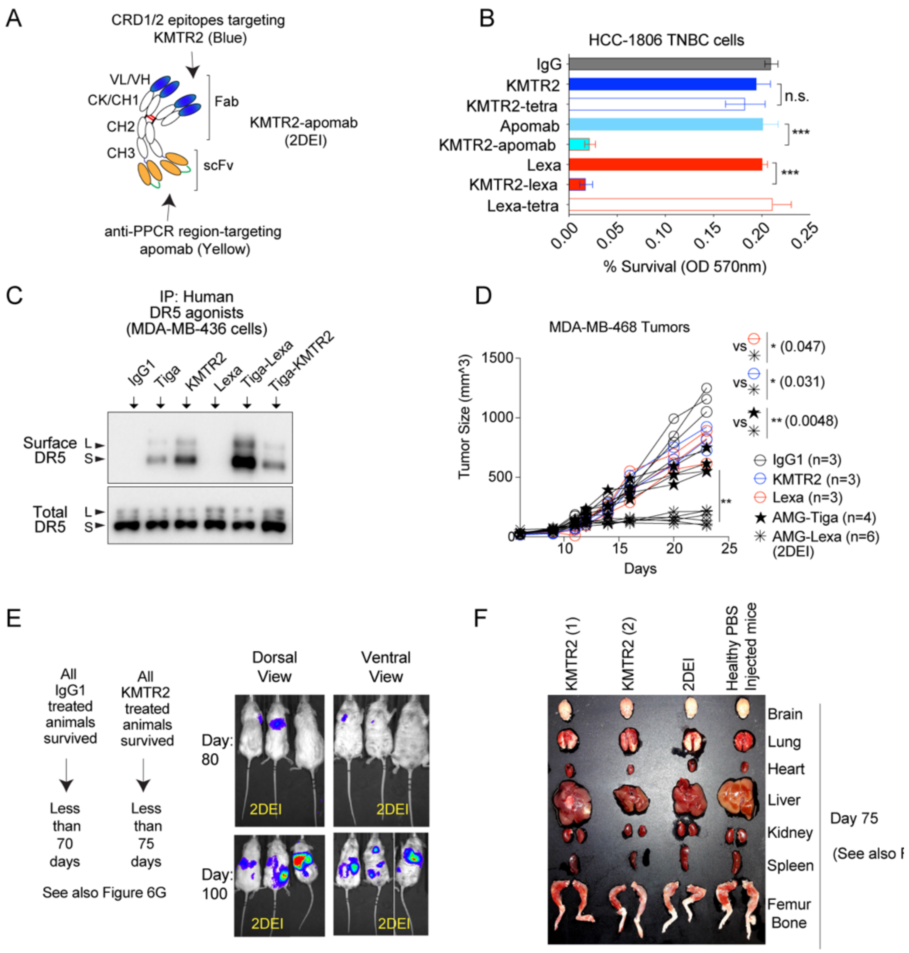

Figure S6. *In-vitro* activity and *in-vivo* function and distribution of clinical DR5 agonists and 2DEI antibody, Related to Figure 5 and 6

(A) Genetic construction schematic of dual specificity 2DEI antibody where CRD1 or CRD2 targeting bispecific partner was engineered with apomab scFv.

(B) The percent survival analysis of HCC-1806 cells in the presence of indicated 2DEI antibodies generated either with Lexa or apomab along with control antibodies.

(C) Native immunoprecipitation of DR5 with indicated DR5 agonists, 2DEI, and random bispecific antibody treatments using MDA-MB-436 cells.

(D) Female NOD.Cg Prkdcscid Il2rgtm1Wjl/SzJ (NSG) mice were grafted with orthotopic tumors of MDA-MB-468 cells subcutaneously. Upon tumor generation, randomly selected animals were i.p. injected with indicated antibodies (100µg) every third day, and tumor volumes were quantified at indicated days by caliper measurements (n=3-6).

(E) NSG animals were grafted to generate primary subcutaneous tumors. When tumors reached ~700mm3(~4 weeks), surgeries were performed to removed tumors. After a 2-week recovery post-surgery, animals were i.p. injected either with IgG1, KMTR2, and 2DEI (100µg) every 3rd day, and tumor-bearing animals were live imaged. Since all IgG1 and KMTR2 treated animals did not survive beyond 75 days, only representative 2DEI treated animals are shown after day 80 and day 100.

(F) Same as D, except animal necropsies were recovered (at day 75) from representative animals and analyzed by fluorescent imaging for detailed organ-specific tumor load. The fluorescent signal is shown in figure 6E.

Error bars in (B) represent SD (n=3).

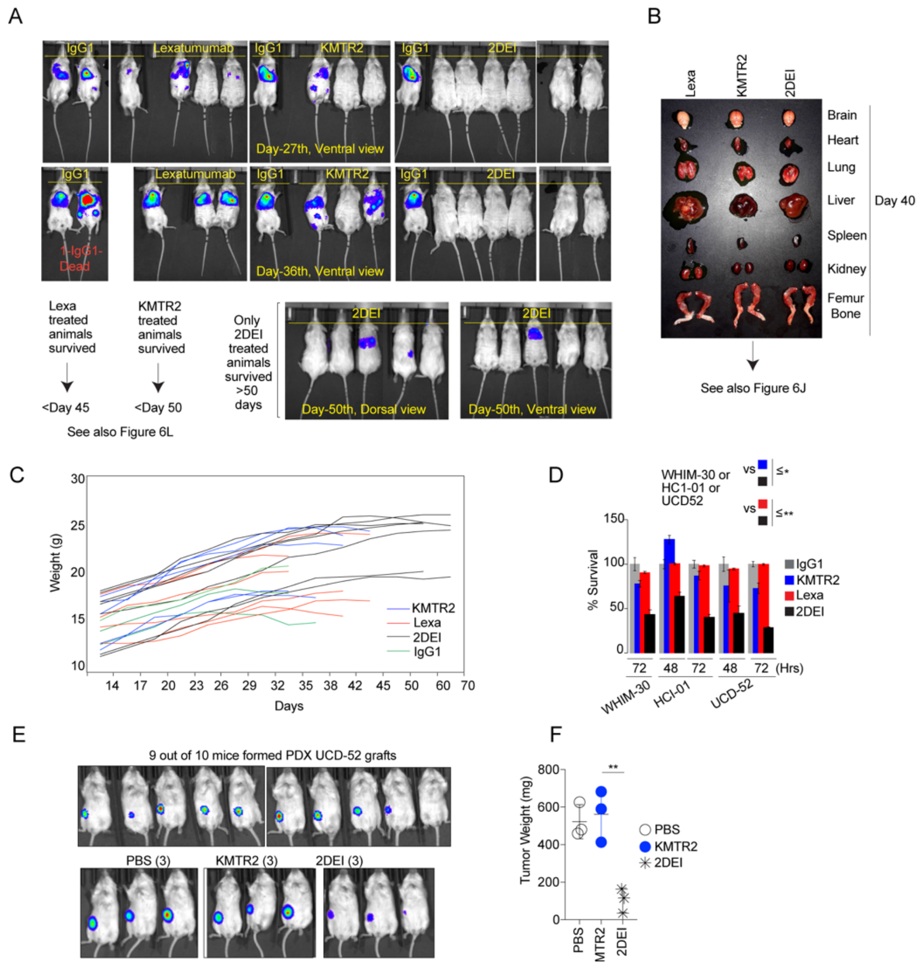

Figure S7. Efficacy of 2DEI antibody in metastatic models and against patient-derived tissue, Related to Figure 6

(A) TNBC brain metastatic derivative of human mammary cells called MDA-MB-231-2B cells (Luc+) were intracardially injected to generate highly aggressive metastatic tumors that spread in the lung, other parts, and peritoneal cavity within 4-5 weeks. Randomly metastatic tumors bearing animals were treated with indicated antibodies (100µg, 2 times a week) and were live imaged at indicated days. A ventral view of representative mice is shown. See Figure 6I for the dorsal view.

(B) Same as A, except animal necropsies from each treatment group were recovered (at day 40) from representative animals and were analyzed by fluorescent imaging for detailed organ-specific tumor burden. The fluorescent signal is shown in figure 6J.

(C) Animals before the generation of experimental metastatic TNBC tumors and during various indicated treatments days were weighed. No significant change in animal weights was observed in 2DEI, and other antibody-treated animals compared to IgG1 treated animals.

(D) Patient-derived breast TNBC UCD52, WHIM-30, HCI-01 tumors cells were tested for cell viability in spheroid cultures in the presence of indicated antibodies. The 2DEI antibody was significantly effective in killing an average of 50% of tumor spheroids within 48 hours, while KMTR2 and lexatumumab were not considerably effective compared to IgG1.

(E) NOD SCID gamma (NSG) mice breast fat pad grafted TNBC PDX UCD52 tumors were treated with either PBS, KMTR2, and 2DEI antibody. After 6 doses, animals were imaged (n=3 mice).

(F) Same as D, except harvested tumor weights were quantified (n=3).

Error bars in (D) and (F) represent SEM and SD respectively (n=3).

DR5 agonists	Sequences
AMG655	VH:<br>QVQLQESGPGLVKPSQTLSLTCTVSGGSISSGDYFWSWIRQLPGKGLEWIGHIHNS<br>GTTYYNPSLKSRVTISVDTSKKQFSLRLSSVTAADTAVYYCARDRGGDYYYGMDV<br>WGQGTTVTVSS<br>VL:<br>EIVLTQSPGTLSLSPGERATLSCRASQGISRSYLAWYQQKPGQAPSLLIYGASSRAT<br>GIPDRFSGSGSGTDFTLTISRLEPEDFAVYYCQQFGSSPWTFGQGTKVEIK
Lexatumumab	VH:<br>EVQLVQSGGGVERPGGSLRLSCAASGFTFDDYGMSWVRQAPGKGLEWVSGINW<br>NGGSTGYADSVKGRVTISRDNAKNSLYLQMNSLRAEDTAVYYCAKILGAGRGWYF<br>DLWGKGTTVTVSS<br>Vl:<br>SSELTQDPAVSVALGQTVRITCQGDSLRSYYASWYQQKPGQAPVLVIYGKNNRPS<br>GIPDRFSGSSSGNTASLTITGAQAEDEADYYCNSRDSSGNHVVFGGGTKLTVL
KMTR2	VH:<br>QVQLVQSGAEMKKPGASVKVSCKTSGYTFTNYKINWVRQAPGQGLEWMGWMNP<br>DTDSTGYPQKFQGRVTMTRNTSISTAYMELSSLRSEDTAVYYCARSYGSGSYYRD<br>YYYGMDVWGQGTTVTVSS<br>VL:<br>EIVLTQSPATLSLSPGERATLSCRASQSVSSYLAWYQQKPGQAPRLLIYDASNRAT<br>GIPARFSGSGSGTDFTLTISSLEPEDFAVYYCQQRSNWPLTFGGGTKVEIK
Tigatuzumab	VH:<br>EVQLVESGGGLVQPGGSLRLSCAASGFTFSSYVMSWVRQAPGKGLEWVATISSG<br>GSYTYYPDSVKGRFTISRDNAKNTLYLQMNSLRAEDTAVYYCARRGDSMITTDYW<br>GQGTLVTVSS<br>VL:<br>DIQMTQSPSSLSASVGDRVTITCKASQDVGTAVAWYQQKPGKAPKLLIYWASTRHT<br>GVPSRFSGSGSGTDFTLTISSLQPEDFATYYCQQYSSYRTFGQGTKVEIK
Apomab	VH:<br>EVQLVQSGGGVERPGGSLRLSCAASGFTFDDYAMSWVRQAPGKGLEWVSGINW<br>QGGSTGYADSVKGRVTISRDNAKNSLYLQMNSLRAEDTAVYYCAKILGAGRGWYF<br>DYWGKGTTVTVSS<br>VL:<br>SELTQDPAVSVALGQTVRITCSGDSLRSYYASWYQQKPGQAPVLVIYGANNRPSGI<br>PDRFSGSSSGNTASLTITGAQAEDEADYYCNSADSSGNHVVFGGGTKLTVL

Table S1. Sequences of DR5 agonist antibodies used, Related to Figure 1

Recombinant DR5	
DR5 RKCW	ITQQDLAPQQRAAPQQKRSSPSEGLCPPGHHISEDGRDCISCKYGQ DYSTHWNDLLFCLRCTRCDSGEVELSPCTTTRNTVCQCEEGTFREE DSPEMCRKCWTGCPRGMVKVGDCTPWSDIECVHKESG
DR5 RKCY	ITQQDLAPQQRAAPQQKRSSPSEGLCPPGHHISEDGRDCISCKYGQ DYSTHWNDLLFCLRCTRCDSGEVELSPCTTTRNTVCQCEEGTFREE DSPEMCRKCYTGCPRGMVKVGDCTPWSDIECVHKESG
DR5 EECE (3RE)	ITQQDLAPQQRAAPQQKRSSPSEGLCPPGHHISEDGRDCISCKYGQ DYSTHWNDLLFCLRCTRCDSGEVELSPCTTTRNTVCQCEEGTFREE DSPEMCEECETGCPRGMVKVGDCTPWSDIECVHKESG
DR5 EECE (3RA)	ITQQDLAPQQRAAPQQKRSSPSEGLCPPGHHISEDGRDCISCKYGQ DYSTHWNDLLFCLRCTRCDSGEVELSPCTTTRNTVCQCEEGTFREE DSPEMCAACATGCPRGMVKVGDCTPWSDIECVHKESG
DR5 RKAR (C103A)	ITQQDLAPQQRAAPQQKRSSPSEGLCPPGHHISEDGRDCISCKYGQ DYSTHWNDLLFCLRCTRCDSGEVELSPCTTTRNTVCQCEEGTFREE DSPEMCRKARTGCPRGMVKVGDCTPWSDIECVHKESG

Table S2. Sequences of recombinant DR5 ectodomains used, Related to Figure 2, 3, and 7
